# Radical docking–migration: a powerful strategy for difunctionalization of alkenes and alkynes

**DOI:** 10.1039/d5sc08771f

**Published:** 2026-01-20

**Authors:** Zhu Cao, Fushan Chen, Chen Zhu

**Affiliations:** a The Education Ministry Key Laboratory of Resource Chemistry, Joint International Research Laboratory of Resource Chemistry of Ministry of Education, Shanghai Key Laboratory of Rare Earth Functional Materials, Shanghai Frontiers Science Center of Biomimetic Catalysis, Shanghai Normal University Shanghai 200234 China; b Frontiers Science Center for Transformative Molecules, School of Chemistry and Chemical Engineering, State Key Laboratory of Synergistic Chem-Bio Synthesis, Shanghai Key Laboratory for Molecular Engineering of Chiral Drugs, Shanghai Jiao Tong University 800 Dongchuan Road Shanghai 200240 China chzhu@sjtu.edu.cn

## Abstract

Radical-mediated difunctionalization of alkenes and alkynes represents a powerful and efficient strategy for constructing complex molecular architecture from simple unsaturated precursors. The docking–migration approach, which involves the initial addition of a radical species to an alkene or alkyne followed by intramolecular migration of a functional group, has emerged as a versatile and broadly applicable tactic. This review summarizes the achievements in this rapidly developing area, with a focus on the design of bifunctional reagents that enable the simultaneous incorporation of two functional groups *via* radical addition and migration cascades. The scope encompasses various migrating groups, including aryl, heteroaryl, alkynyl, oximino, alkenyl, and imino units, and highlights extensions to challenging unactivated alkenes and alkynes. The mechanistic insights, substrate scope, and synthetic applications of these transformations are discussed, underscoring the potential of the radical docking–migration strategy in modern synthetic chemistry.

## Abstract 2

This review highlights recent advances in radical-mediated alkene and alkyne difunctionalization, leveraging a docking–migration strategy with novel bifunctional reagents.

## Graphical abstract

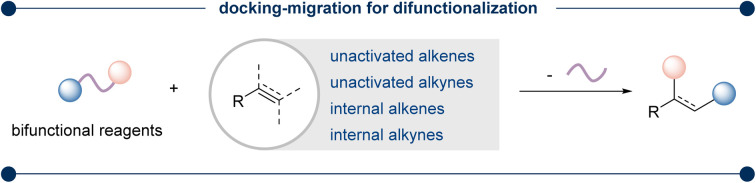

## 1 Introduction

**Scheme 1:**
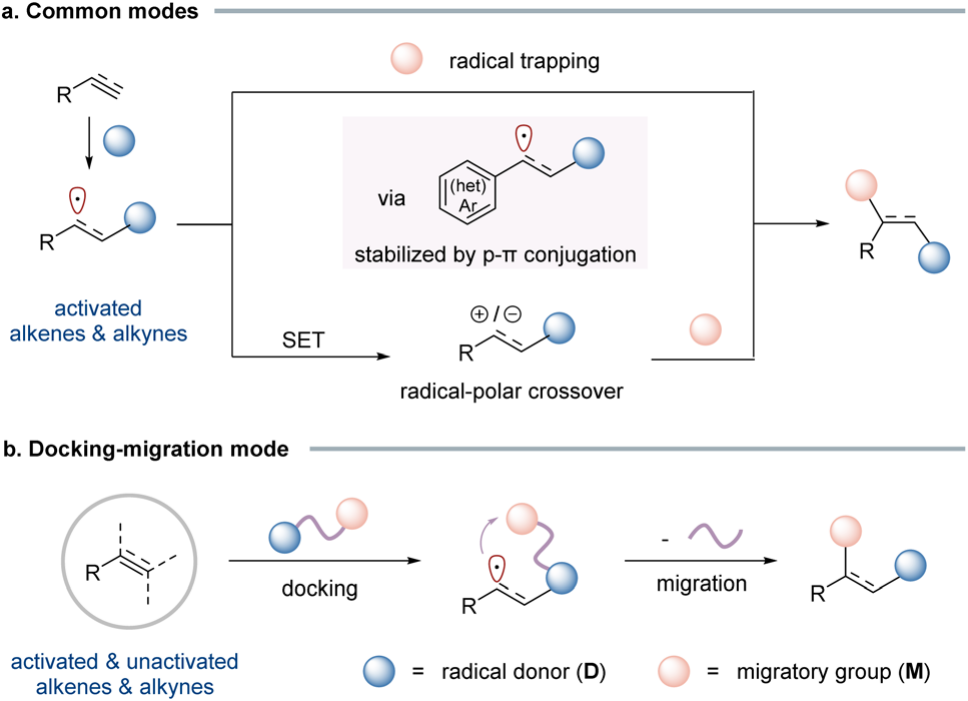
Radical-mediated difunctionalization of alkenes and alkynes.

Alkenes and alkynes are among the most fundamental and versatile building blocks in organic synthesis, enabling the construction of complex molecular architecture through a wide array of functionalization reactions. Radical-mediated difunctionalization offers a powerful and step-economic approach to simultaneously introduce two functional groups across unsaturated carbon–carbon bonds, thereby rapidly increasing molecular complexity from simple precursors.^1^ Despite significant advancements over the past several decades, the current synthetic methods remain largely constrained to activated alkenes that feature a neighbouring π-system, such as an aryl, carbonyl, or heteroatom, capable of stabilizing the transient alkyl radical intermediate through p–π conjugation or heteroatom lone pair–π interactions (Scheme 1a). Conversely, the difunctionalization of unactivated alkenes, including simple aliphatic olefins, continues to pose substantial challenges.

Radical-mediated difunctionalization of alkynes to access polysubstituted alkenes remains relatively underdeveloped.^2^ This can be attributed to well-recognized kinetic and thermodynamic challenges,^3^ including the high reactivity of vinyl radical intermediates generated upon addition of extrinsic radicals to alkynes. These species often undergo undesirable rapid cyclization or addition to other π-systems, in addition to participating in fast intramolecular or intermolecular hydrogen atom transfer (HAT) processes. As a result, contemporary synthetic approaches still rely heavily on the use of activated alkynes, exemplified by phenylacetylene derivatives, where the resulting vinyl radical intermediates are stabilized through p–π conjugation. By contrast, unactivated aliphatic alkynes, which lack such a stabilizing effect, present significant hurdles. Furthermore, free radical-mediated alkyne difunctionalization frequently yields alkene products as mixtures of *Z*/*E* isomers, thereby limiting the synthetic utility of these methods.^4^

To address these challenges, a breakthrough was achieved with the development of the “docking–migration” strategy, pioneered by our group.^5,6^ Bifunctional reagents, bearing a radical donor (D) and a migratory group (M) connected *via* a linker, are readily synthesized in a few steps. The radical addition (docking) of D to the alkene/alkyne generates a radical intermediate that triggers the subsequent intramolecular migration of M (Scheme 1b). This strategically designed sequence effectively converts an intermolecular reaction into an intramolecular process, ensuring efficient difunctionalization with broad substrate compatibility. This strategy offers several distinct advantages over traditional three-component approaches. (1) It significantly expands the substrate scope, enabling the difunctionalization of challenging unactivated alkenes and alkynes. These substrates are often recalcitrant in conventional intermolecular radical processes due to polarity mismatches, unfavorable kinetics, or competing side reactions. (2) The intramolecular migration step proceeds through a cyclic transition state, which often imparts exceptional control over stereochemistry. Remarkably, for cycloalkenes, this renders kinetic control of stereoselectivity to generate the non-trivial *cis*-products. (3) By merging two coupling partners into a single, well-designed bifunctional reagent, the docking–migration approach enhances synthetic efficiency, minimizes byproduct formation, and improves atom economy.

This review summarizes the accomplishments in the radical-mediated difunctionalization of alkenes and alkynes *via* the docking–migration strategy. We discuss the development of various bifunctional reagents, featuring sulfone, tertiary alcohol, ether, sulfoximine, and ester structural motifs, which enable aryl, heteroaryl, alkynyl, oximino, alkenyl, and imino migrations. Notably, we also introduce the asymmetric docking–migration processes, which have unlocked novel, enantioselective pathways for alkene difunctionalization. The scope encompasses both activated and unactivated substrates, highlighting the versatility of this approach for the synthesis of complex molecules. Furthermore, we extend the discussion to alkyne difunctionalization, where functional group migrations have recently been applied to achieve stereo- and regio-selective transformations that were previously considered challenging.

## 2 Alkene difunctionalization

### 2.1 (Hetero)Aryl migration

**Scheme 2:**
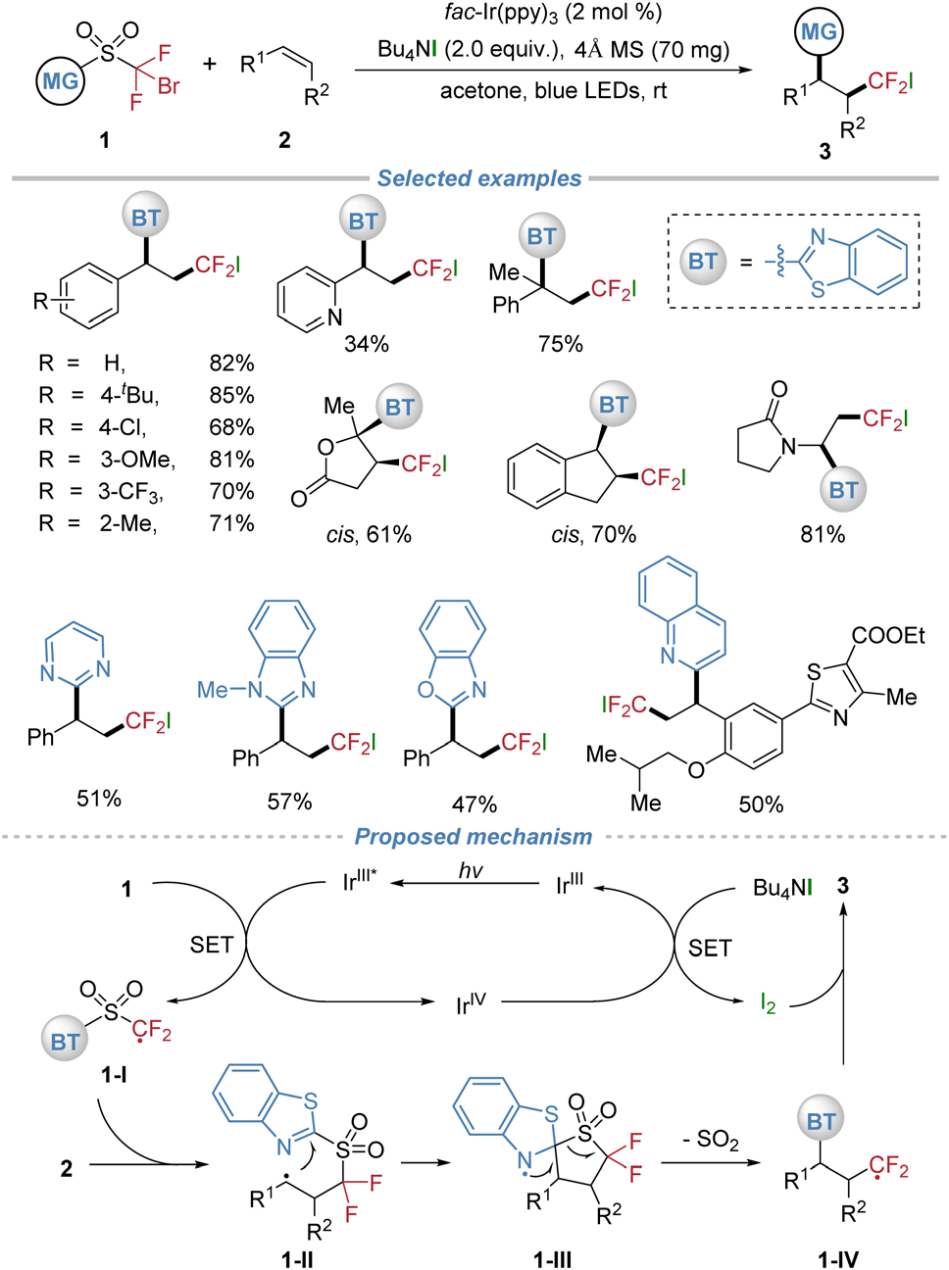
Difluoromethylative heteroarylation of alkenes *via* docking–migration.

In 2018, our group established the concept of radical docking–migration, achieving the photocatalytic difluoromethylative heteroarylation of alkenes using sulfone-based bifunctional reagents 1 (Scheme 2).^6^ This method enables the efficient intermolecular fluoroalkyl radical addition followed by intramolecular heteroaryl migration, delivering valuable heteroaryl substituted alkyl difluorides 3. Both activated and unactivated alkenes underwent smooth transformation under mild photocatalytic conditions, demonstrating broad substrate generality. The tetrabutylammonium iodide (TBAI) additive played a dual role as a reductant to turn over the photocatalytic cycle and as the halogen source incorporated into the final product. The design of the bifunctional reagents was crucial, allowing diverse N-containing heteroarenes, including benzothiazole, pyrimidine, and quinoline, to be efficiently installed *via* the docking–migration process. Furthermore, the protocol was successfully applied to the late-stage functionalization of complex drug molecules and natural product derivatives, highlighting its potential for the rapid synthesis of structurally diverse compounds.

In the proposed mechanism, the Ir^III^ photocatalyst is promoted to its excited state with visible light irradiation, which undergoes oxidative quenching by the C–Br bond of substrate 1. This step affords the difluoromethyl radical 1-I along with an Ir^IV^ species. The resultant radical 1-I adds across the alkene, yielding the alkyl radical intermediate 1-II. Subsequently, this alkyl radical is captured intramolecularly by the benzothiazolyl group *via* a five-membered cyclic transition state, forming the spiro N-radical species 1-III. Extrusion of SO_2_ then delivers the difluoroalkyl radical 1-IV. Concurrently, single-electron transfer from TBAI to the Ir^IV^ species regenerates the ground-state Ir^III^ catalyst and liberates iodine, which subsequently couples with radical 1-IV to furnish the final difunctionalized product 3. This sequence perpetuates the catalytic cycle, ensuring continuous turnover.

**Scheme 3:**
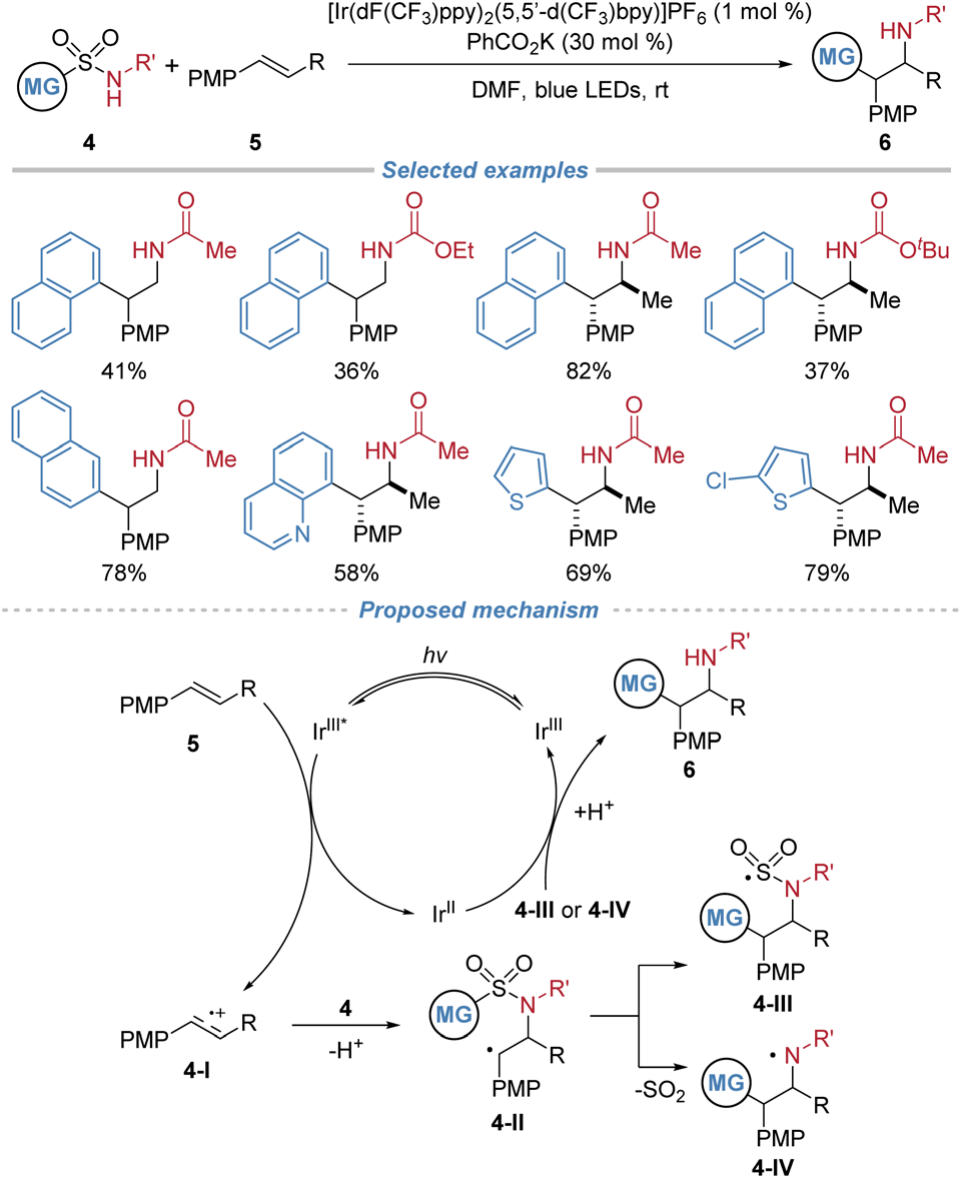
Arylsulfonylacetamides as bifunctional reagents for the difunctionalization of electron-rich alkenes.

In the same year, Stephenson *et al.* reported a visible light induced aminoarylation of electron-rich alkenes using arylsulfonylacetamides 4 as bifunctional reagents, enabling the synthesis of 2,2-diarylethylamines with high *anti*-Markovnikov regioselectivity and good diastereoselectivity (Scheme 3).^7^ This redox-neutral transformation proceeds *via* a radical cation intermediate 4-I generated through single-electron oxidation of an electron-rich alkene, followed by nucleophilic addition of 4 to generate intermediate 4-II and a subsequent Smiles type 1,4-aryl migration to produce intermediate 4-III or 4-IV. Sequential interaction with the photocatalyst yields product 6, while regenerating the photocatalyst. Conducted under mild conditions with concomitant release of SO_2_, the reaction exhibits good functional group compatibility. However, the scope of applicable alkenes and bifunctional reagents is largely limited.

**Scheme 4:**
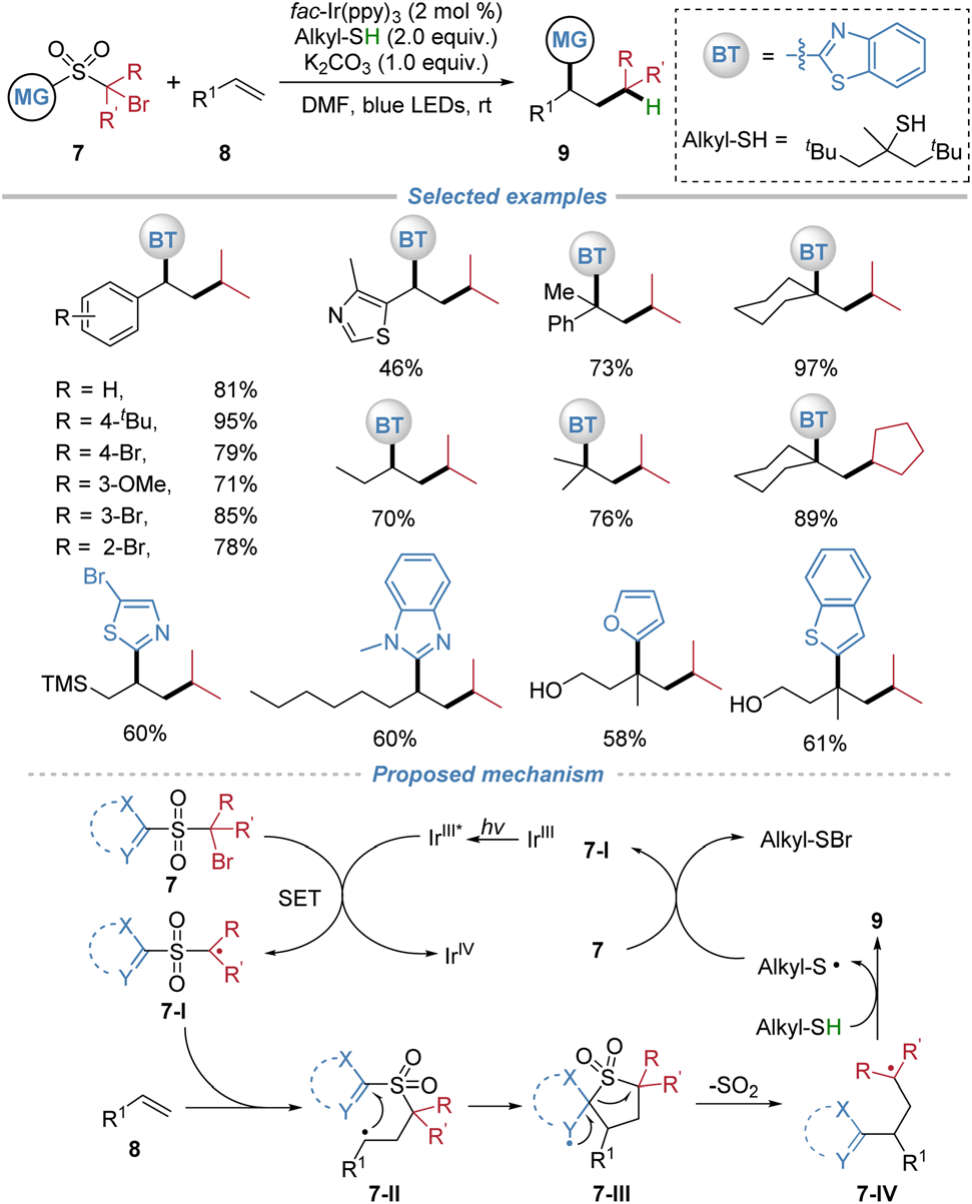
Polarity-umpolung radical heteroaryl–alkylation of aliphatic alkenes.

The installation of a sulfonyl group could invert the inherent polarity of an alkyl radical, rendering it electrophilic and thereby enabling addition to electron-neutral or rich aliphatic alkenes. Following radical addition, the ensuing docking–migration cascade with concomitant SO_2_ extrusion would ultimately regenerate the original alkyl group, thereby achieving a long-standing challenging radical alkylation of unactivated alkenes. In 2020, our group reported the radical alkylation of aliphatic alkenes by the docking–migration strategy using sulfone-based bifunctional reagents 7 (Scheme 4).^8^ This strategy enables the concomitant introduction of an alkyl group and a heteroaryl moiety across a broad range of alkenes. The key design involves the generation of electrophilic sulfone-bearing alkyl radicals, thereby overcoming the polarity mismatch problem that typically hinders the radical alkylation of unactivated alkenes. Under mild photoredox conditions using *fac*-Ir(ppy)_3_ as a photocatalyst and *tert*-dodecylthiol as both a hydrogen atom donor and reductant, the reaction proceeds efficiently to afford a wide range of valuable alkylated products. The method exhibits excellent functional group tolerance, accommodating sensitive functionalities such as free alcohol, epoxide, and aldehyde. Notably, the protocol is applicable to complex natural products and drug molecules, enabling late-stage functionalization. The proposed mechanism involves photoredox-induced generation of an electrophilic alkyl radical 7-I from the bifunctional reagents. This radical adds to the alkene, forming intermediate 7-II, which undergoes intramolecular heteroaryl or oximino migration *via* a cyclic transition state. Subsequent extrusion of SO_2_ yields radical intermediate 7-IV, which is then reduced by the thiol to furnish the final product and regenerate the thiyl radical, thereby sustaining the catalytic cycle.

**Scheme 5:**
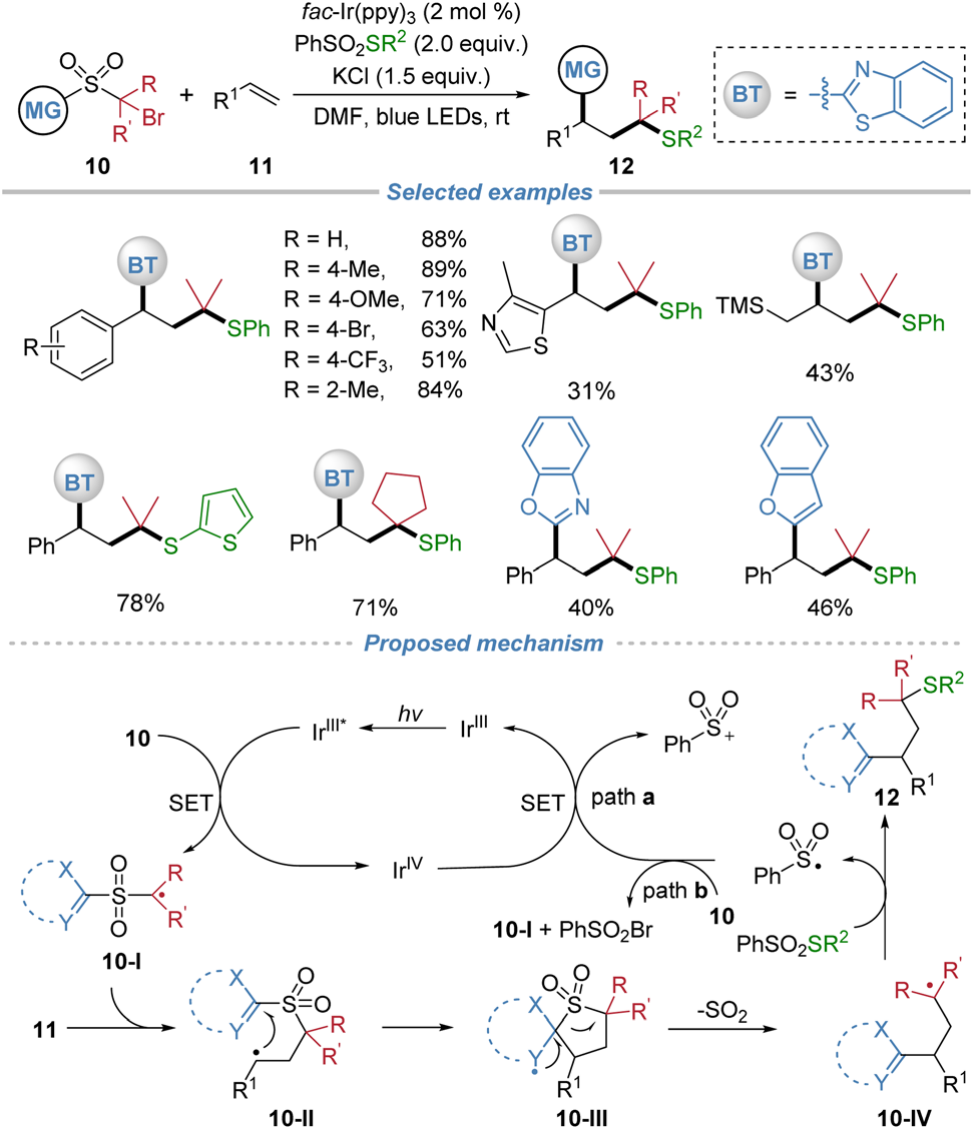
Polarity-umpolung three-component radical alkylation of alkenes.

This polarity-umpolung protocol was extended to three-component radical alkylation of alkenes (Scheme 5).^9^ This method simultaneously introduced a thioalkyl and a heteroaryl group across alkene. The reaction proceeds under mild photoredox conditions with *fac*-Ir(ppy)_3_ as the catalyst and exhibits broad functional group tolerance, accommodating a wide range of styrenes, aliphatic alkenes, and complex alkene molecules. Key to the success is also the rational design of the sulfone-based bifunctional reagents, which initially generates an electrophilic alkyl radical 10-I under photoreduction conditions. This radical adds regioselectively to the alkene, followed by intramolecular heteroaryl migration and SO_2_ extrusion to form a new alkyl radical intermediate 10-IV. This radical is then efficiently trapped by phenyl benzenethiosulfonate (PhSO_2_SPh) or analogous reagents, yielding the corresponding heteroaryl-substituted alkyl thioethers.

**Scheme 6:**
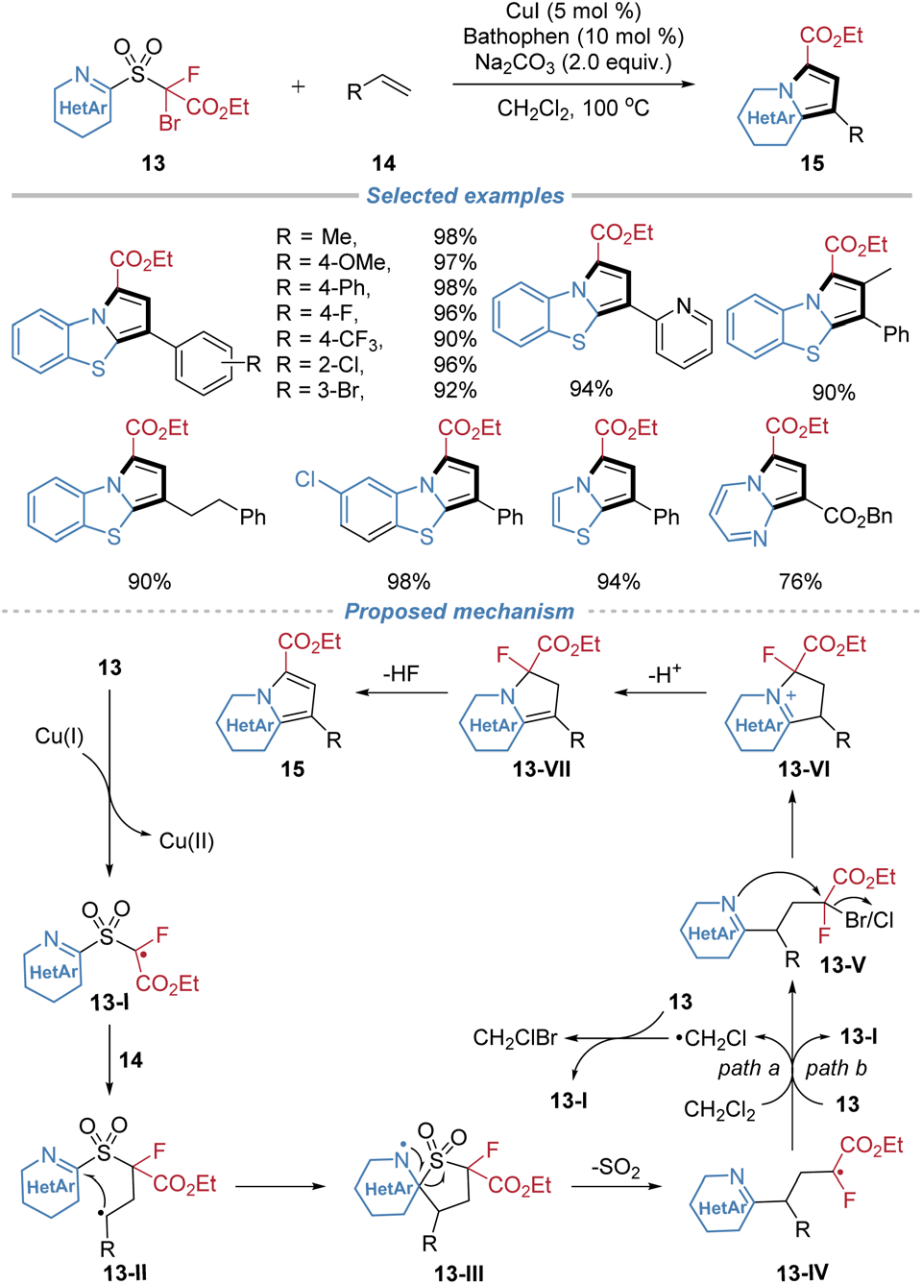
Assembly of heteroarenes *via* the docking–migration–cyclization cascade.

In 2021, our group reported the assembly of N-fused heteroarenes from alkenes using sulfone-based heterocyclizing reagents 13 (Scheme 6).^10^ This method leverages the docking–migration process, followed by an intramolecular cyclization, to efficiently construct valuable heterocyclic scaffolds. Operating under simple copper catalysis, the transformation initiates *via* a single-electron transfer to generate an electrophilic alkyl radical 13-I from the reagent. This radical adds regioselectively across the alkene substrate, triggering an intramolecular heteroaryl migration with concomitant SO_2_ extrusion to form a new alkyl radical intermediate 13-IV, which then abstracts a halogen atom from the solvent or the bifunctional reagent to form intermediate 13-V. This adduct simultaneously undergoes intramolecular cyclization caused by a nucleophilic nitrogen atom from the migrated heteroaryl group, finally furnishing the N-fused heterocyclic product. The protocol accommodates a wide range of activated and unactivated alkenes with broad functional group tolerance. Furthermore, the modular design of the bifunctional reagents enables access to seven distinct types of N-fused heterocyclic cores.

**Scheme 7:**
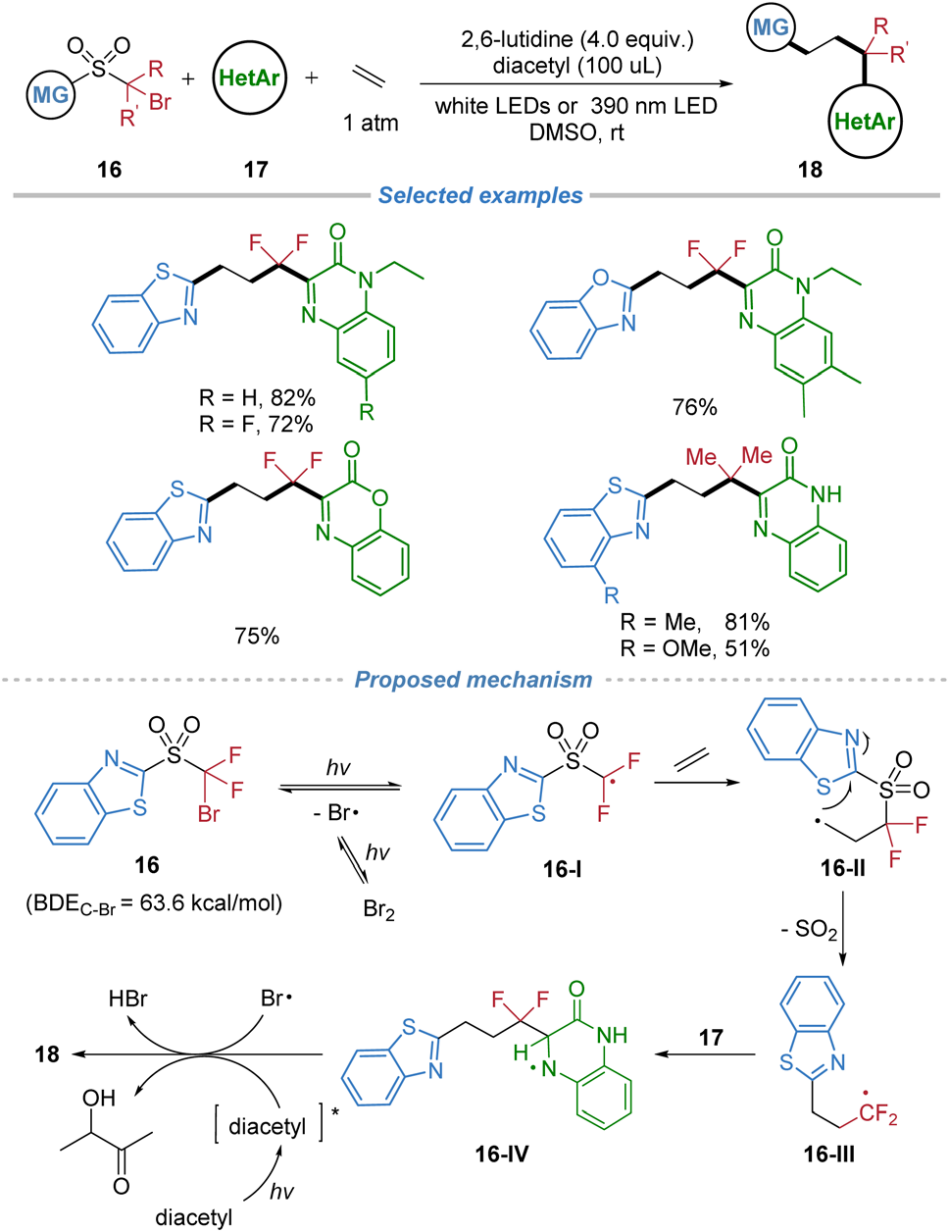
Three-component radical 1,2-difunctionalization of ethylene.

A metal-free radical difunctionalization of ethylene was disclosed in 2023 (Scheme 7).^11^ Operating under mild photocatalytic conditions, the method leverages a functional group migration strategy to achieve three-component heteroarylation of ethylene. The process begins with photolytic homolysis of the C–Br bond in the sulfone reagent, generating an electrophilic carbon radical 16-I that preferentially adds to ethylene over electron-deficient heteroarenes. The resulting primary alkyl radical intermediate 16-II undergoes rapid intramolecular migration of a heteroaryl group *via* a kinetically favored five-membered transition state, followed by SO_2_ extrusion to form a nucleophilic radical 16-III. This species is then trapped by a variety of N-heteroarenes in a Minisci-type reaction, delivering densely functionalized heteroarylated products with high selectivity and broad functional group tolerance. The strategy effectively suppresses competing ethylene oligomerization or polymerization, highlighting the power of radical docking–migration processes for the difunctionalization of the simplest alkene under metal-free conditions.

**Scheme 8:**
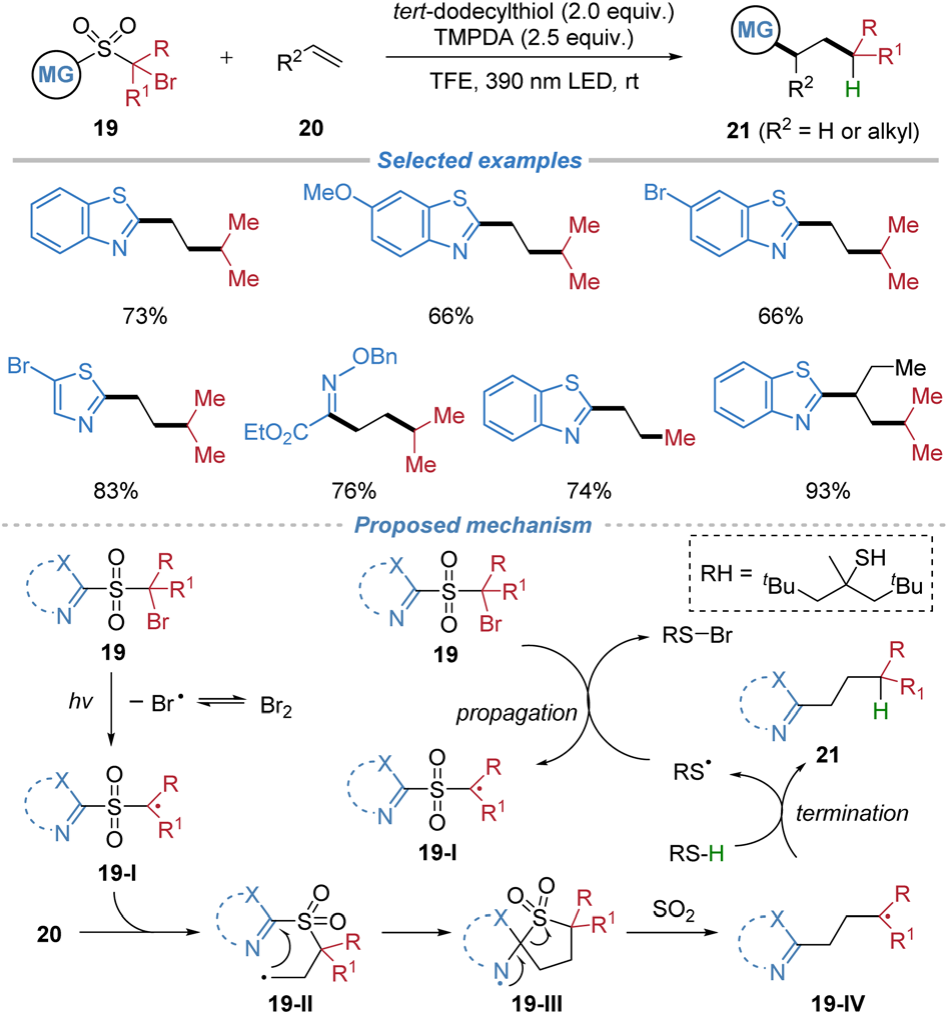
Radical alkyl–heteroaryl difunctionalization of low-boiling-point alkenes.

This protocol was further extended to a metal-free alkylheteroarylation of ethylene and other low-boiling-point alkenes using rationally designed sulfone-based bifunctional reagents 19 (Scheme 8).^12^ The process is initiated by the visible-light-induced homolytic cleavage of a C–Br bond, generating an electrophilic alkyl radical 19-I that adds to ethylene. This addition is followed by an intramolecular migration of a heteroaryl or oximino group *via* a kinetically favored five-membered transition state, with concomitant extrusion of SO_2_ to form a nucleophilic radical 19-IV. The resulting radical is then trapped by hydrogen atom transfer from a thiol, delivering diverse heteroaryl/oximino-substituted alkane products. Notably, the reaction proceeds efficiently without external photocatalysts or transition metals and is applicable to a range of gaseous and low-boiling-point alkenes. The method also allows for deuterium incorporation *via* D_2_O.

**Scheme 9:**
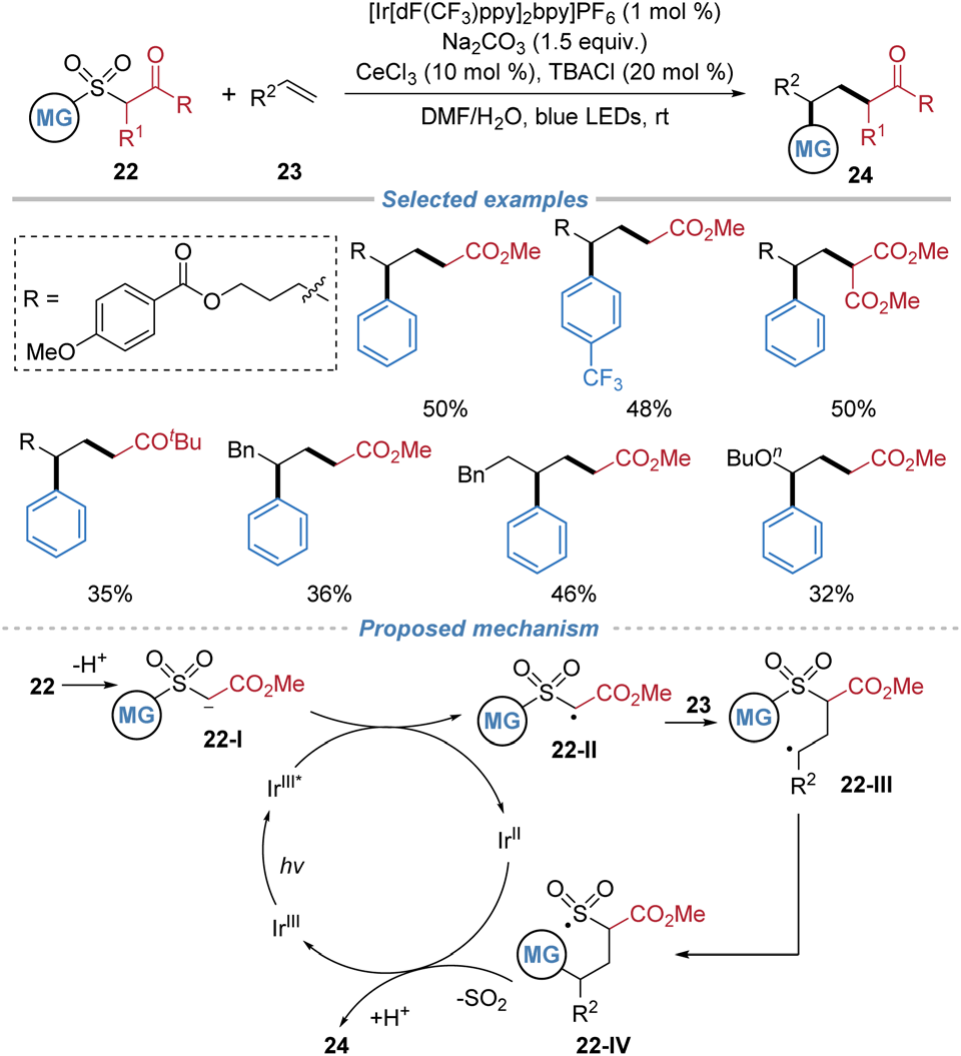
Arylsulfonyl acetates as bifunctional reagents for alkene difunctionalization.

In 2023, Hong *et al.* reported a photocatalytic desulfonylative carbonylation of alkenes using arylsulfonyl acetates 22 as bifunctional reagents (Scheme 9).^13^ In the presence of an Ir-based photocatalyst, the deprotonated sulfonyl acetate species undergoes single-electron oxidation to generate an electrophilic α-carbonyl radical 22-II. This radical adds across an alkene to form a carbon-centered adduct 22-III, which then undergoes intramolecular aryl migration *via* a Smiles type rearrangement, culminating in desulfonylation and simultaneous formation of two C–C bonds. This method allows the efficient incorporation of both aryl and ester groups across unactivated alkenes, providing direct access to valuable γ-aryl ester derivatives under mild photochemical conditions.

**Scheme 10:**
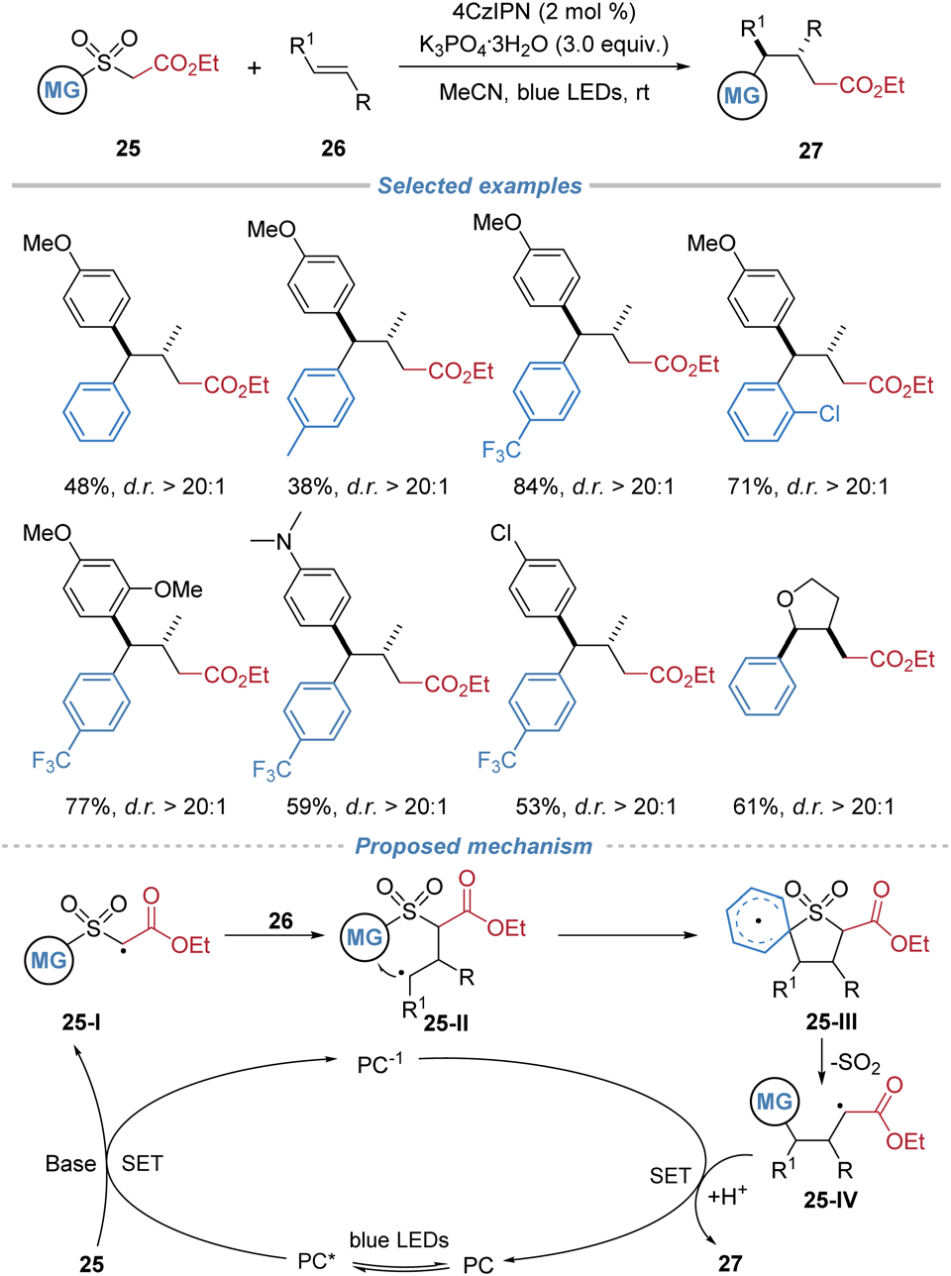
Arylsulfonyl acetates as bifunctional reagents for the difunctionalization of internal alkenes.

Liu *et al.* also disclosed the photoredox-catalytic alkylarylation of internal alkenes employing arylsulfonyl acetates 25 as bifunctional reagents (Scheme 10).^14^ Under blue light irradiation with 4CzIPN as the photosensitizer, the deprotonated sulfonyl acetate species undergoes single-electron oxidation to generate an electrophilic α-carbonyl radical. This radical adds across an alkene to form a carbon-centered adduct, which then undergoes an intramolecular Smiles rearrangement, leading to desulfonylation and simultaneous formation of C–C bonds. This cascade affords valuable γ-aryl substituted ester derivatives with high regio- and diastereo-selectivity and broad functional group tolerance. In the proposed mechanism, single-electron oxidation of the deprotonated arylsulfonyl acetate by the photoexcited 4CzIPN catalyst generates an electrophilic α-carbonyl radical 25-I, which regioselectively adds to the alkene, forming a benzylic radical intermediate 25-II. Subsequent intramolecular *ipso*-radical addition *via* a Smiles rearrangement, facilitated by a favorable π–π stacking interaction, delivers a cyclic spiroradical intermediate 25-III. Fragmentation of this intermediate then results in extrusion of SO_2_ and formation of a stabilized alkyl radical 25-IV. Finally, single-electron reduction and protonation afford the ester product.

**Scheme 11:**
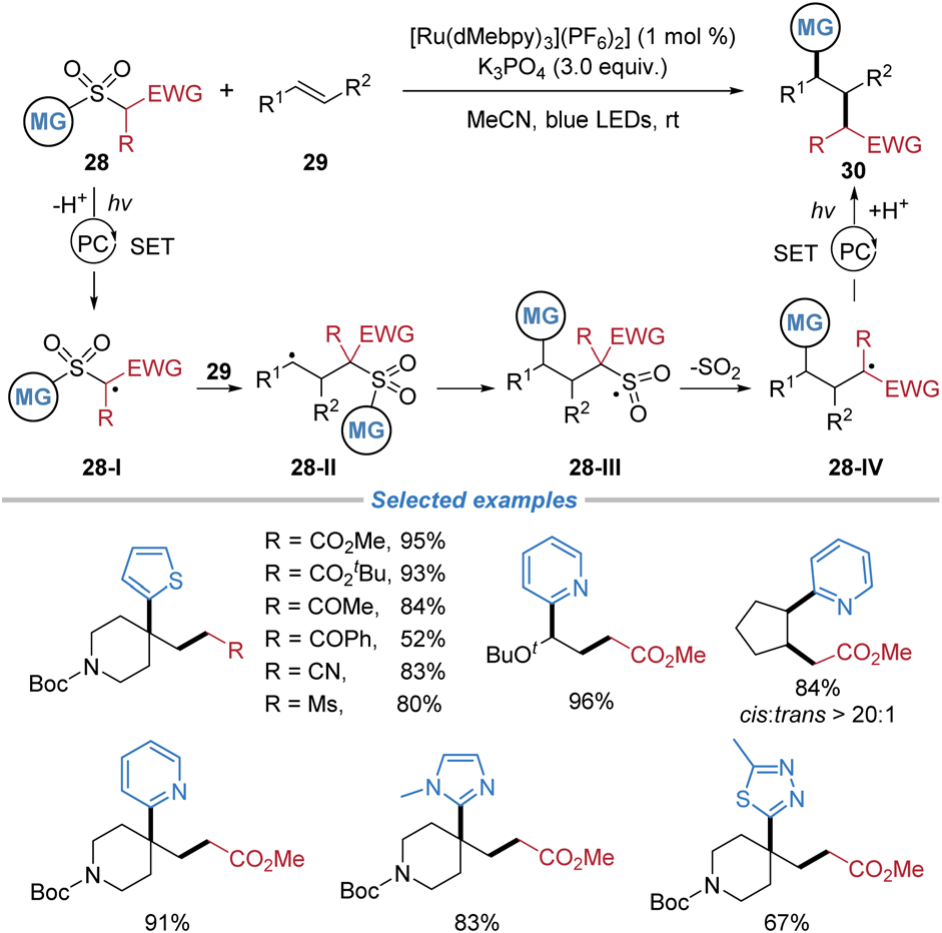
Radical alkyl–(hetero)arylation of alkenes.

In 2024, Nacsa *et al.* reported a similar alkyl–(hetero)arylation of alkenes using alkyl–(hetero)aryl sulfones 28 as bifunctional reagents under photoredox conditions (Scheme 11).^15^ Key to the process is the photoinduced generation of an electrophilic alkyl radical 28-I from the sulfone reagent, which adds to the alkene to form a new C–C bond. The resulting carbon-centered radical 28-II undergoes an intramolecular 1,4-(hetero)aryl migration *via* a radical Smiles rearrangement, followed by desulfonylation to forge the second C–(hetero)aryl bond. This method accommodates mono- to tetra-substituted alkenes, exhibits excellent diastereoselectivity in both cyclic and acyclic systems, and allows for the incorporation of primary and secondary alkyl groups, as well as N-heteroarenes.

**Scheme 12:**
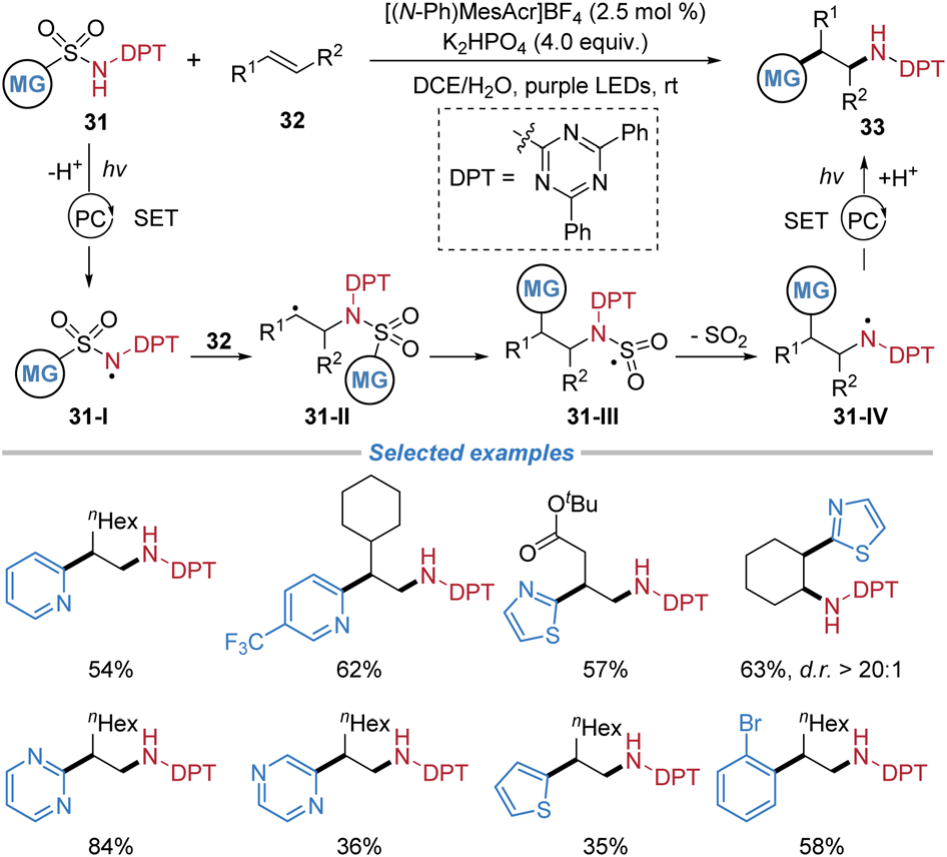
Radical amino–(hetero)arylation of unactivated alkenes.

Later, the same group disclosed a radical amino–(hetero)arylation of alkenes employing (hetero)aryl sulfonamides 31 equipped with an *N*-diphenyl-2,4,6-triazinyl (DPT) protecting group (Scheme 12).^16^ This strategy overcomes chemoselectivity challenges associated with the oxidative instability of primary amino–(hetero)arylation products and the inherent decomposition pathways of *N*-acyl sulfonamidyl radicals prevalent in conventional protected systems. Under photoredox conditions utilizing a highly oxidizing acridinium-type photocatalyst, deprotonation and single-electron oxidation of the *N*-DPT sulfonamide generates an electrophilic sulfonamidyl radical 31-I. This nitrogen-centered radical adds across a broad range of unactivated simple alkenes, from mono- to trisubstituted, forging the initial C–N bond. The resultant alkyl radical 31-II subsequently undergoes a desulfonylative radical Smiles rearrangement, facilitating a 1,4-(hetero)aryl migration to establish the key C–(hetero)aryl bond and deliver the protected amino–(hetero)arylation product. The rationally designed *N*-DPT group not only prevents product decomposition by raising its oxidation potential but also avoids the deleterious fragmentation that plagues carbonyl-based protecting groups, thereby enabling the successful radical cascade. The reaction exhibits excellent functional group tolerance, good diastereoselectivity, and accommodates a wide array of N-heteroarenes, including pyridine, pyrimidine, and thiazole.

**Scheme 13:**
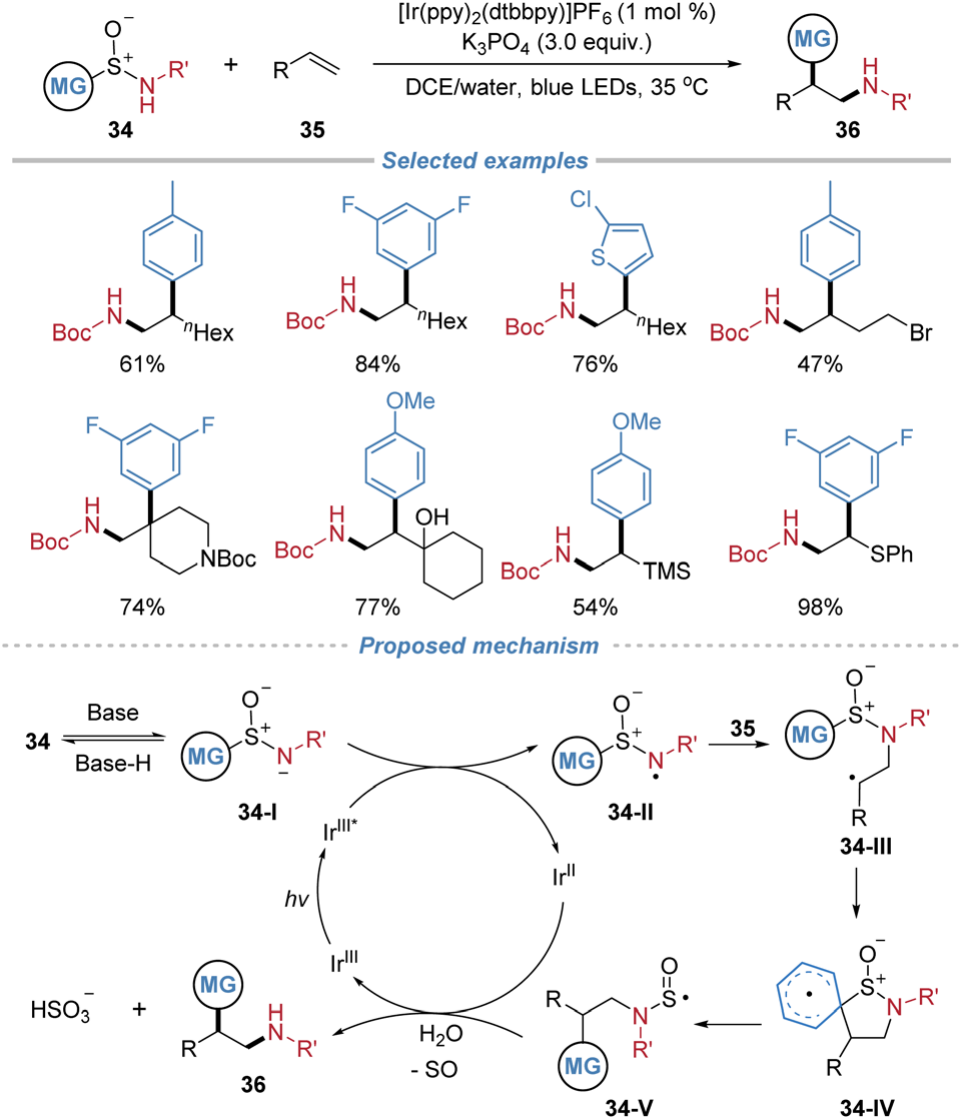
Aryl sulfinamides as bifunctional reagents for alkene difunctionalization.

In 2024, Stephenson *et al.* introduced aryl sulfinamides as bifunctional reagents 34 for the radical aminoarylation of alkenes (Scheme 13).^17^ Operating under mild photocatalytic conditions with a weakly oxidizing photocatalyst, the method enables the generation of a nitrogen-centered radical that adds to alkenes, forming C–N bonds. This initiation is followed by a desulfinative Smiles rearrangement, which facilitates aryl migration and forges a new C–C bond. The process proves applicable to a variety of unactivated alkenes. The transformation commences with the base-assisted deprotonation of sulfinamide 34, yielding anionic species 34-I. Subsequent single-electron oxidation converts 34-I into nitrogen-centered radical 34-II, which undergoes radical addition across the alkene to furnish intermediate 34-III. Radical adduct 34-III then participates in a Smiles rearrangement *via* a dearomatized spirocyclic intermediate 34-IV. Subsequent rearomatization facilitates homolytic cleavage of the C–S bond, generating *N*-sulfinyl radical 34-V. This species may either undergo direct homolytic dissociation or be reductively cleaved by the reduced-state photocatalyst, ultimately releasing SO and affording the corresponding amidyl radical or anion, which is protonated to yield the final product.

**Scheme 14:**
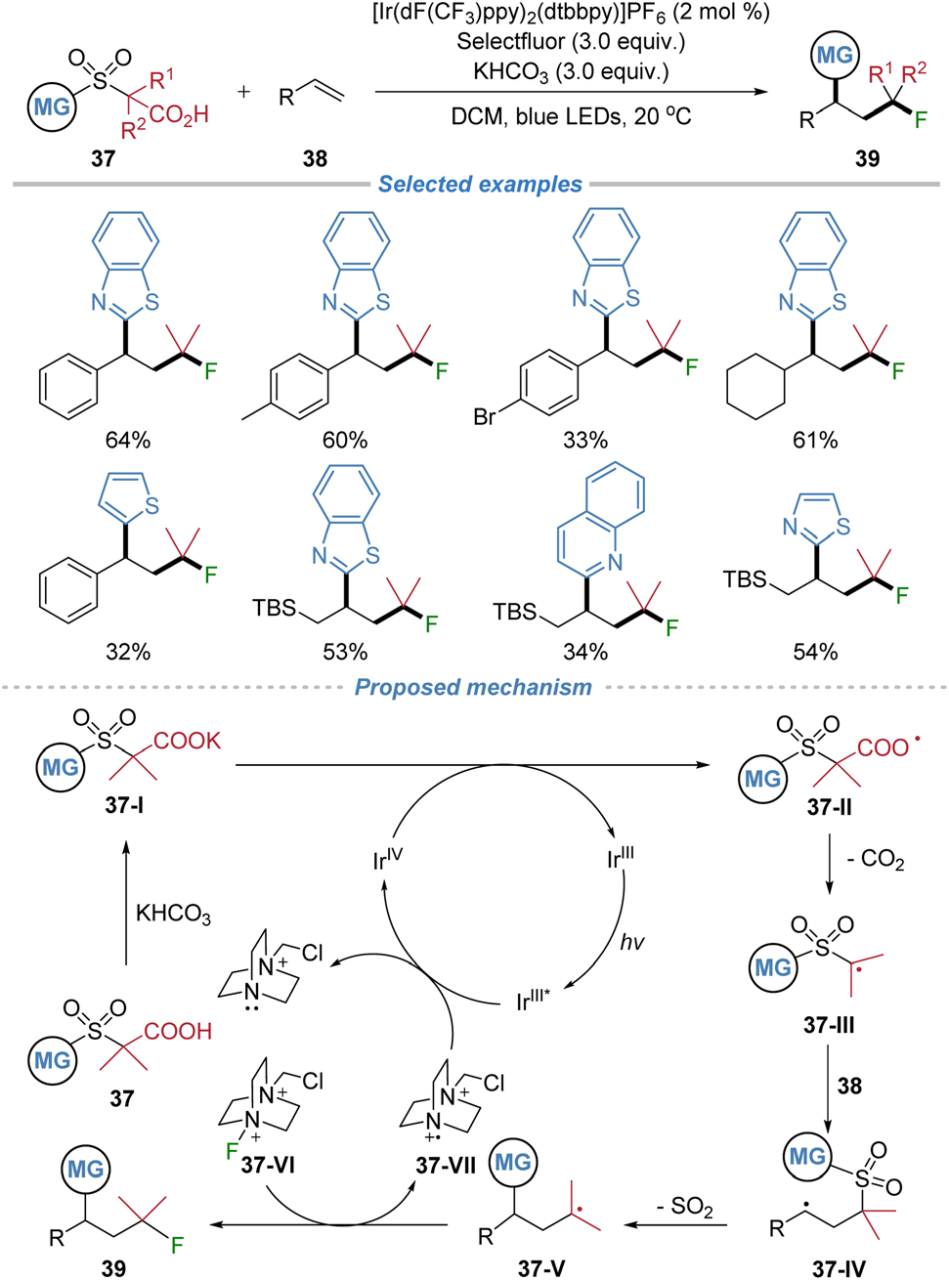
Modular synthesis of alkyl fluorides *via* decarboxylative–desulfonylative difunctionalization of alkenes.

In 2025, our group reported the modular synthesis of alkyl fluorides *via* a radical decarboxylative–desulfonylative difunctionalization of alkenes (Scheme 14).^18^ Employing α-sulfonyl carboxylic acids 37 as bifunctional reagents under Ir- based photoredox catalysis, the method enables the generation of alkyl radicals 37-III*via* decarboxylation. These radicals add to both styrenes and aliphatic alkenes, forming a C–C bond. The resulting adduct undergoes a kinetically favored intramolecular heteroaryl migration *via* a radical Smiles rearrangement, accompanied by SO_2_ extrusion, to forge a second C–C bond. The nucleophilic alkyl radical intermediate then abstracts a fluorine atom from Selectfluor to yield the final product. This process concurrently constructs two C–C bonds and one C–F bond with broad substrate scope, including complex molecules, highlighting the power of radical docking–migration cascades to access valuable fluorinated building blocks.

**Scheme 15:**
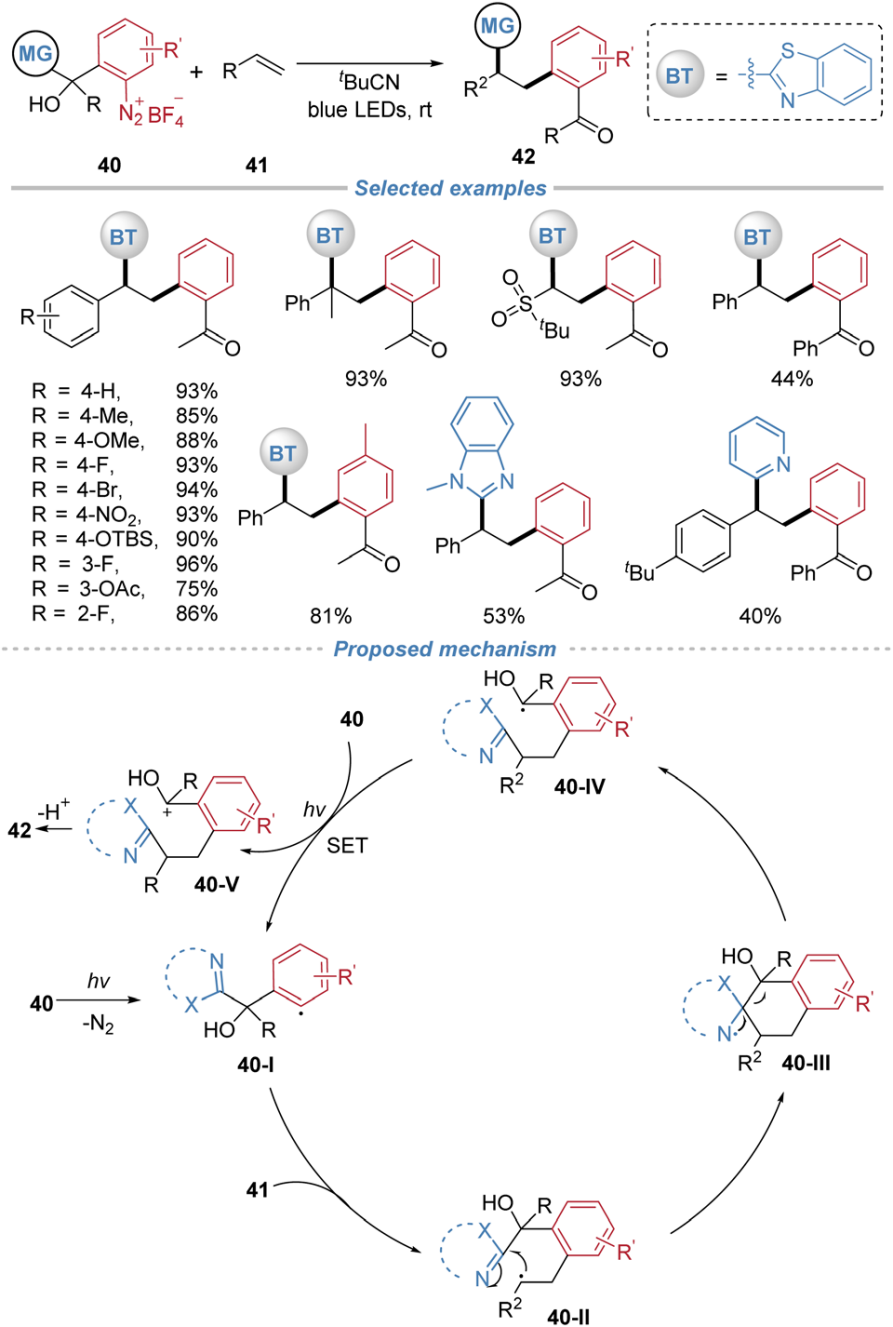
Radical-mediated 1,2-arylheteroarylation of alkenes with tertiary alcohol-based aryldiazonium salts as bifunctional reagents.

In 2021, our group disclosed a catalyst-free 1,2-arylheteroarylation of alkenes *via* the radical docking–migration process involving the cleavage of an inert C–C bond (Scheme 15).^19^ The reaction utilizes tertiary alcohol-based aryldiazonium salts 40 as bifunctional reagents, without the need for any external photocatalyst or additives. The transformation is initiated by photoinduced homolysis of the diazonium salt, generating an aryl radical 40-I that adds across the alkene. The resulting alkyl radical intermediate 40-II then undergoes an intramolecular 1,5-heteroaryl migration, accompanied by cleavage of a stable C(sp^3^)–C(sp^3^) bond, to form a ketyl radical species 40-IV. This reductive ketyl radical subsequently donates an electron to another molecule of the aryldiazonium salt, regenerating the aryl radical and perpetuating a radical chain cycle. Termination *via* deprotonation yields the valuable polyarylalkane product. The protocol tolerates styrenes and electron-deficient alkenes. The strategic design of the bifunctional reagent, which incorporates both the migrating heteroaryl group and the cleavable tertiary alcohol moiety, is crucial for enabling this cascade.

**Scheme 16:**
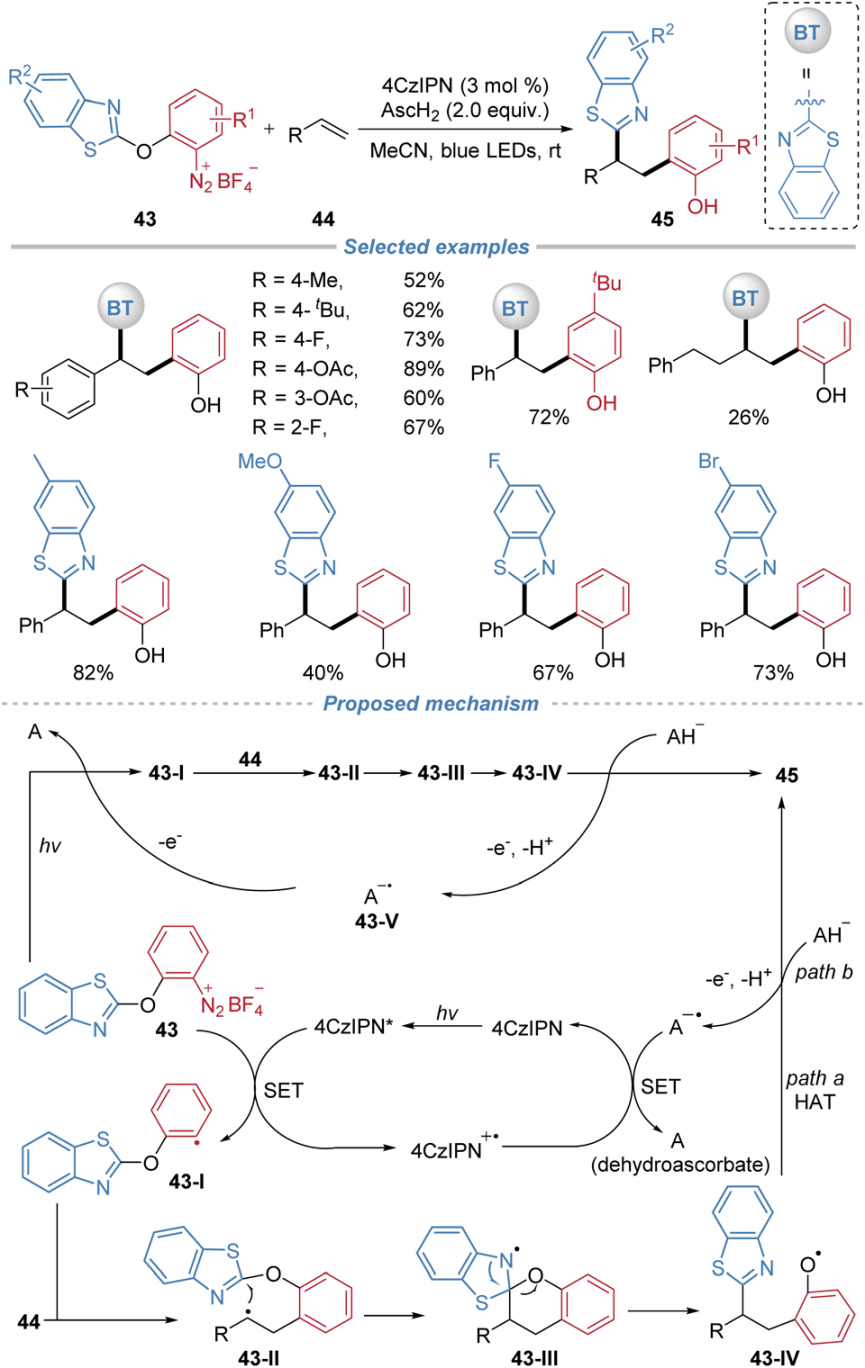
Intermolecular radical difunctionalization by docking–migration of arylbenzothiazolylether reagents.

Subsequently, our group reported a photocatalytic carboarylation of alkenes *via* the cleavage of the inert C–O bond in diaryl ethers (Scheme 16).^20^ This method employs arylbenzothiazolylether-based diazonium salts 43 as bifunctional reagents, 4CzIPN as an organic photocatalyst, and ascorbic acid (AscH_2_) as a reductant. The transformation begins with the photo-reduction of the diazonium salt to generate an aryl radical 43-I, which adds across the alkene to form an alkyl radical 43-II. The resulting alkyl radical intermediate 43-II then adds intramolecularly to the benzothiazolyl group. This addition triggers a 1,5-heteroaryl migration, which concomitantly cleaves the C(Ar)–O bond and generates the phenoxy radical 43-IV. The H-abstraction by 43-IV from ascorbic acid results in the corresponding carboarylation product (path a). Alternatively, radical 43-IV can be reduced by the ascorbate monoanion to the corresponding phenolate, which undergoes subsequent protonation to afford product 45 (path b). The radical chain process may be initiated *via* one of two pathways: either the direct photolytic homolysis of 43 to afford aryl radical 43-I under visible light, or the reduction of 43 to 43-I by 43-V. Subsequently, both pathways converge in the same docking–migration cascade to yield product 45. The reaction is compatible with both activated and unactivated alkenes. The products feature two valuable handles: a phenolic OH group amenable to cross-coupling and a benzothiazolyl moiety that serves as a masked aldehyde, enabling extensive post-synthetic diversification.

**Scheme 17:**
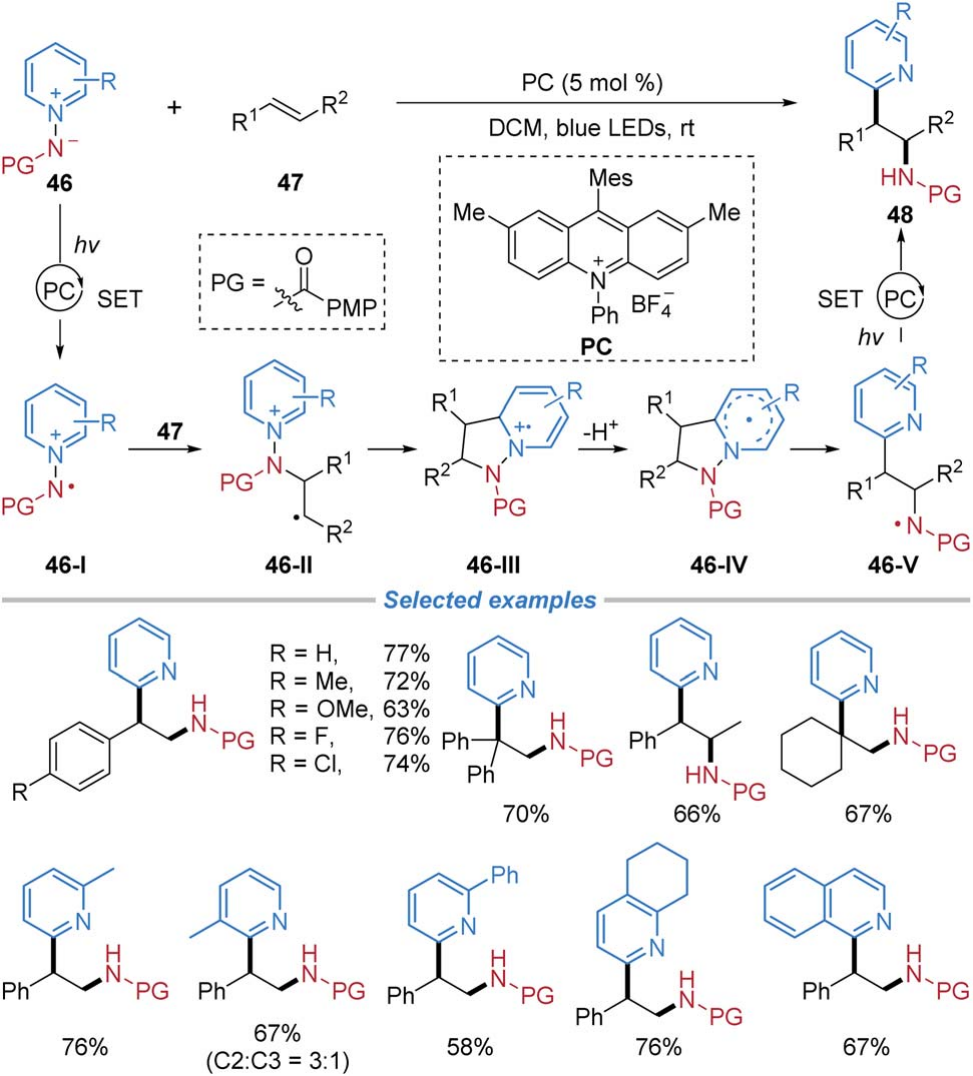
*Ortho*-Selective aminopyridylation of alkenes with *N*-aminopyridinium ylides.

*N*-Aminopyridinium ylides, which serve as precursors for generating amidyl radicals, have been successfully employed as bifunctional reagents for alkene difunctionalization. In 2020, Hong *et al.* reported the radical aminopyridylation of alkenes with C-2 regioselectivity employing *N*-aminopyridinium ylides 46 (Scheme 17).^21^ A wide range of substituted pyridinium ylides proved compatible with the method, each affording the desired products in synthetically useful yields. This approach was amenable to styrenes and unactivated alkenes. Furthermore, internal alkenes were successfully employed, delivering *syn*-adducts with high diastereoselectivity. Mechanistically, *N*-aminopyridinium ylides participate in single-electron oxidation with a photoexcited catalyst to generate pyridinium radical cation 46-I. This reactive intermediate undergoes 1,3-dipolar cycloaddition with an alkene, yielding cycloadduct 46-III. Following deprotonation and *N*–*N* bond cleavage, a reductive SET event concludes the sequence, leading to *ortho*-selective aminopyridylation of alkenes.

**Scheme 18:**
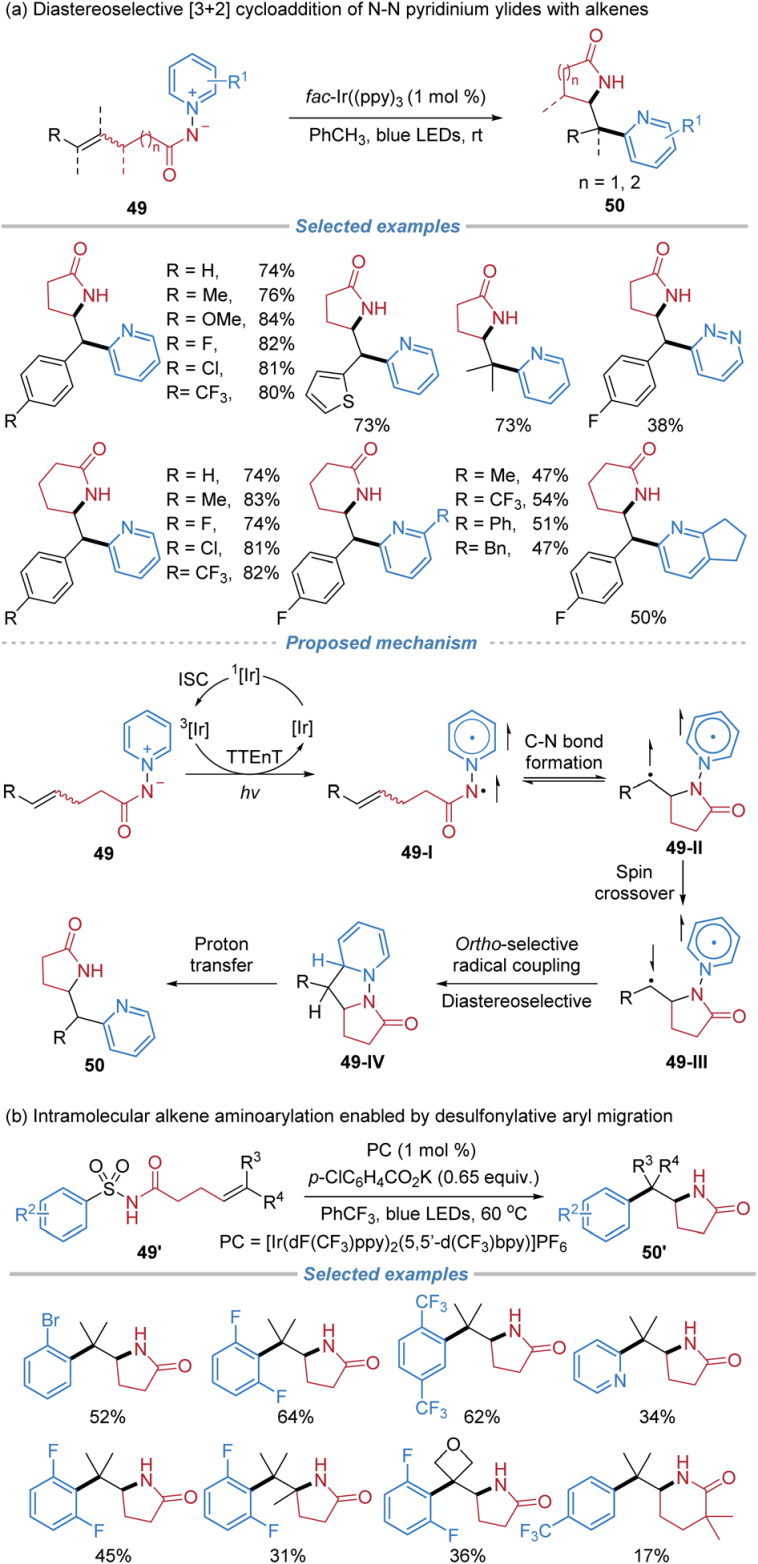
Radical docking–migration strategies for alkene aminoarylation.

Later, the same group reported a diastereoselective pyridyl lactamization enabled by a photo-mediated [3 + 2] cycloaddition with *N*-aminopyridinium ylides 49 as the bifunctional reagents, involving an intramolecular docking–migration process (Scheme 18a).^22*a*^ This protocol exhibits high efficiency and exclusive *syn*-diastereoselectivity, providing direct access to *ortho*-pyridyl-substituted γ- and δ-lactams in a single step. A proposed mechanism involves triplet–triplet energy transfer from a photocatalyst to an *N*–*N* pyridinium ylide species 49. Upon irradiation, energy transfer from photoexcited *fac*-Ir(ppy)_3_ to 49 populates the triplet state of the pyridinium ylide 49-I. The resulting amidyl radical engages in intramolecular addition to a tethered alkene on the triplet potential energy surface, forming a C–N bond and generating a diradical intermediate 49-II. Subsequent intersystem crossing and radical recombination at the *ortho*-position of the pyridinium ring lead to a dearomatized species 49-IV, which upon proton transfer affords the final lactam product. The diastereoselectivity ratio (d.r.) is excellent primarily because the reaction proceeds *via* a stepwise, triplet diradical intermediate in a stereoconvergent manner. Regardless of the initial *E*/*Z* configuration of the alkene, the cyclization occurs through a favored conformation that minimizes steric repulsion between the substituents on the alkene and the lactam backbone.

In a related yet mechanistically distinct approach, Stephenson *et al.* developed a photocatalytic intramolecular aminoarylation of unactivated alkenes using *N*-acylsulfonamides 49′ as bifunctional reagents (Scheme 18b).^22*b*^ This transformation proceeds *via* a nitrogen-centered radical generated through a sequential deprotonation and single-electron oxidation under photoredox conditions. The resulting sulfonamidyl radical undergoes 5-*exo-trig* cyclization onto a tethered alkene, followed by a desulfonylative 1,4-aryl migration to deliver lactam 50′. The method tolerates a broad range of substrates, offering complementary access to structurally diverse, densely functionalized lactam frameworks under mild conditions without the need for transition-metal cross-coupling catalysts.

### 2.2 Asymmetric (hetero)aryl migration

**Scheme 19:**
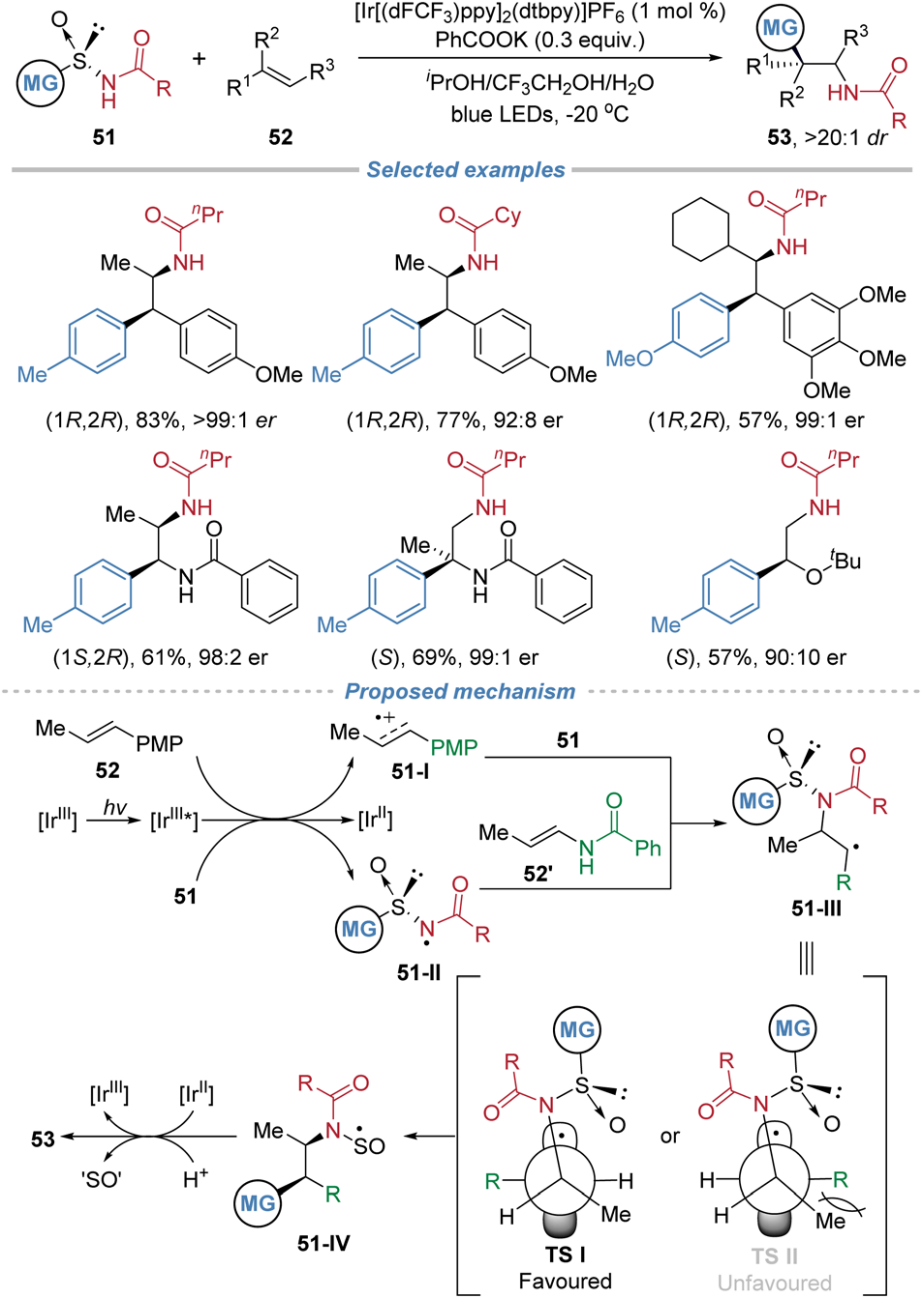
Asymmetric radical aminoarylation of alkenes using chiral arylsulfinamides as bifunctional reagents.

In 2024, Nevado *et al.* reported a photocatalytic asymmetric aminoarylation of alkenes using chiral arylsulfinylamide 51 as bifunctional reagents (Scheme 19).^23^ This strategy leverages the sulfinyl group as a traceless chiral auxiliary to install amino and aryl groups simultaneously across the π-system with exceptional regio- and stereo-control. The process initiates through single-electron oxidation of the electron-rich alkene or the deprotonated sulfinamide, depending on the substrate. The resulting nitrogen-centered radical 51-II adds regioselectively to the terminal position of disubstituted alkenes to form a C–N bond. The subsequent radical-mediated 1,4-aryl migration *via* a Smiles rearrangement forges the C–C bond, delivering enantiomerically enriched β,β-diarylethylamines, aryl-α,β-ethylenediamines, or α-aryl-β-aminoalcohols. The sulfinyl group is cleaved *in situ* under the reaction conditions, rendering the chiral auxiliary traceless. The method exhibits broad functional group tolerance and high stereoselectivity, enabling the construction of two contiguous stereocenters in a single step. The *d*.*r*. is excellent because the reaction employs a chiral arylsulfinylamide reagent where the sulfinyl group acts as a traceless chiral auxiliary. This auxiliary controls the absolute stereochemistry during C–N bond formation. The subsequent 1,4-aryl migration proceeds through a stereospecific, conformationally restricted transition state, minimizing competing pathways. Additionally, the reaction is performed at low temperature, further enhancing stereocontrol. Mechanistic studies, including Stern–Volmer quenching experiments and DFT calculations, support a dual initiation pathway and elucidate the origin of stereocontrol, which is governed by the chiral sulfoxide during the radical addition step.

**Scheme 20:**
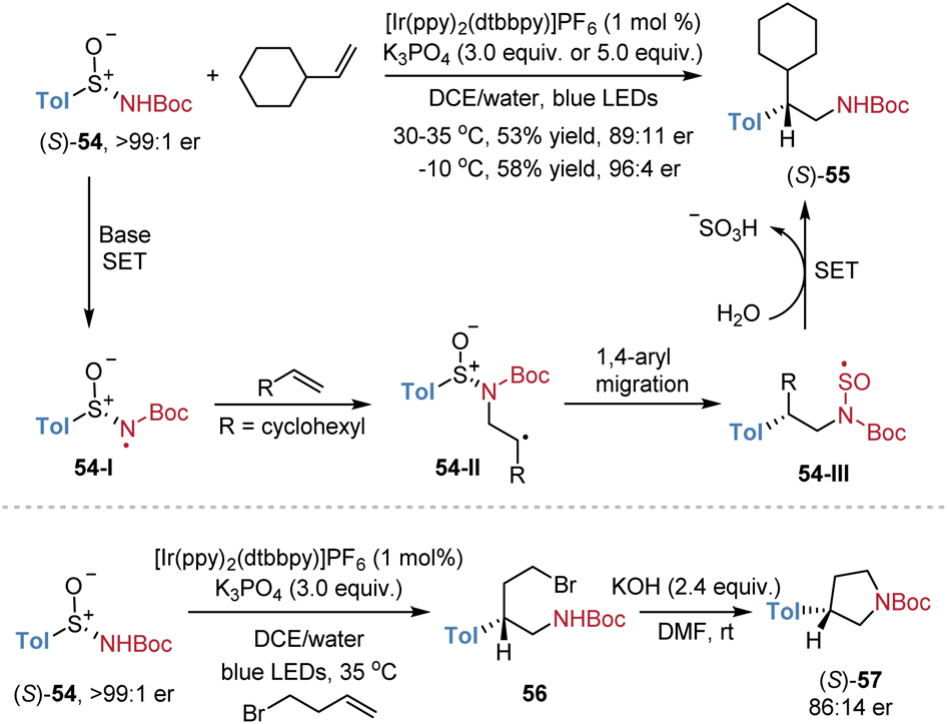
Asymmetric radical aminoarylation of alkenes.

Stephenson *et al.* reported a similar example of asymmetric radical aminoarylation of alkenes employing chiral aryl sulfinamides 54 as bifunctional reagents (Scheme 20).^17^ The reaction of enantiopure (*S*)-54 with vinylcyclohexane afforded the corresponding product (*S*)-55 in 53% yield with 89 : 11 er. When the temperature was lowered to 0 °C, the product was obtained in an improved yield of 58% and with higher stereoselectivity (96 : 4 er). Meanwhile, the reaction of (*S*)-54 with 4-bromobutene yielded the enantioenriched product 56, which underwent subsequent cyclization to give pyrrolidine (*S*)-57 in 86 : 14 er. However, the authors only investigated two examples, with a mechanism analogous to that in Scheme 13.

**Scheme 21:**
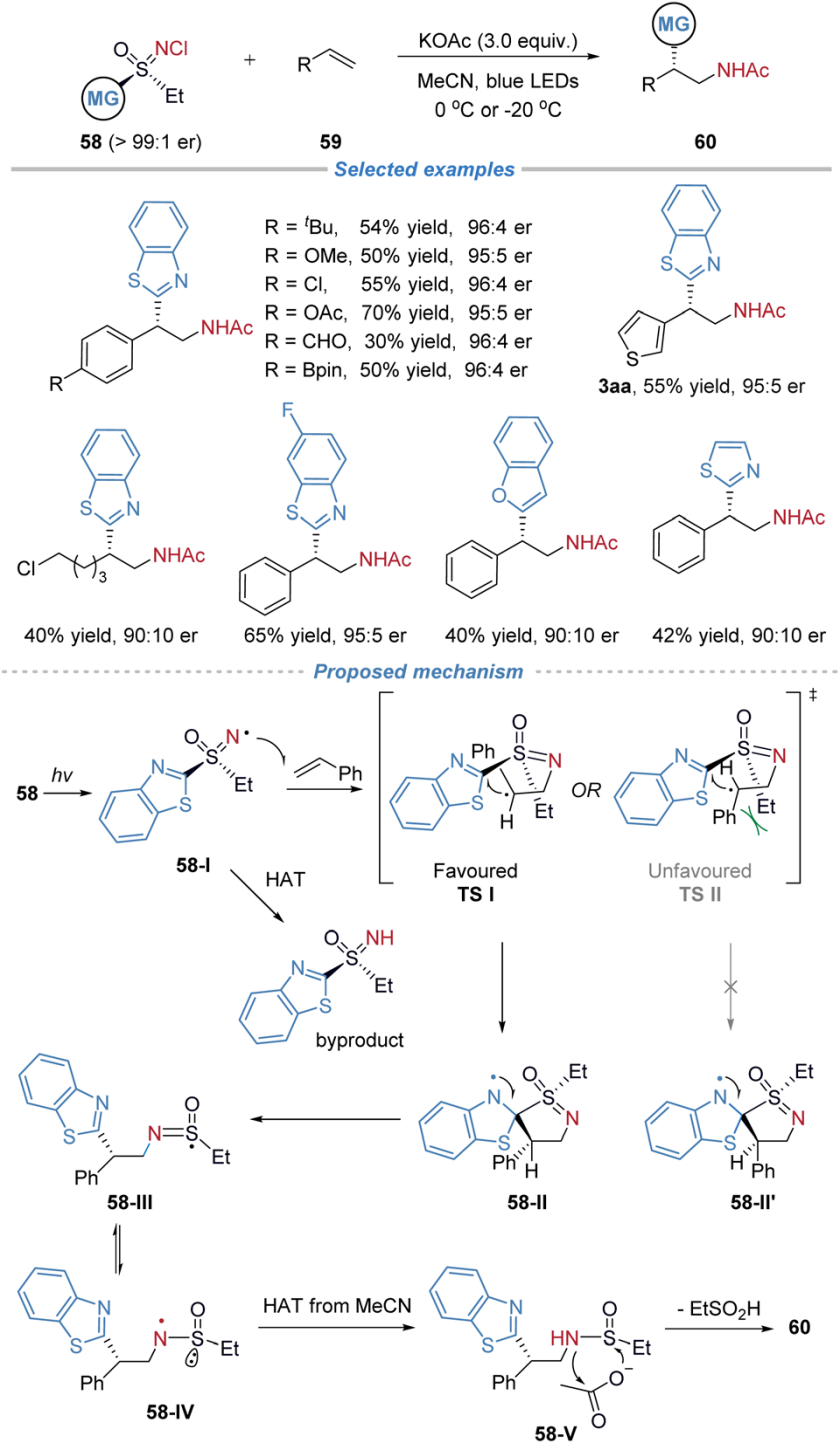
Asymmetric radical aminohetarylation of alkenes with chiral sulfoximine reagents.

In 2024, our group disclosed the asymmetric amino(hetero)arylation of alkenes employing chiral sulfoximines 58 as bifunctional reagents (Scheme 21).^24^ The bifunctional reagents were readily synthesized in three steps: asymmetric oxidation of the thioether to the sulfoxide, followed by further oxidation to the sulfoximine and subsequent chlorination. This sequence afforded the products in good yields and facilitated gram-scale production. This strategy enables the preparation of enantioenriched β-(hetero)arylethylamines without the need for transition-metal catalysts or additional photocatalysts. The method exhibits broad functional group tolerance, accommodating sensitive motifs such as boronic ester, aldehyde, and free alcohol, and proves applicable to the late-stage functionalization of complex molecules. Preliminary mechanistic studies, including radical trapping and cyclopropane ring-opening experiments, support a radical docking–migration pathway, while deuterium labelling confirms the role of the acetate base as the acetyl source in the final product. In the proposed mechanism, *R*-58 undergoes homolytic cleavage of the N–Cl bond under visible-light irradiation, generating the *N*-centered radical intermediate 58-I. This radical adds to styrene, forming an alkyl radical that subsequently undergoes intramolecular cyclization with the benzothiazolyl group to afford spiro intermediate 58-II. Alternatively, radical 58-I may participate in a hydrogen atom transfer process to yield the N–H precursor, which can be recovered and reconverted into the sulfoximine reagent. The C–C bond formation occurs with *S*-configuration *via* the favored transition state TS-I; the TS-II is disfavored due to steric repulsion between the phenyl and ethyl groups. The ensuing heteroaryl migration is a stereospecific step that transfers the chirality from the sulfur center to the carbon backbone with high fidelity, concurrently establishing the new C–C bond and leading to sulfur-centered radical 58-III. This radical then tautomerizes to sulfinamidyl radical 58-IV, which abstracts a hydrogen atom from the solvent to give species 58-V. Finally, 58-V reacts with potassium acetate to deliver the desired product *S*-60.

### 2.3 Alkynyl migration

**Scheme 22:**
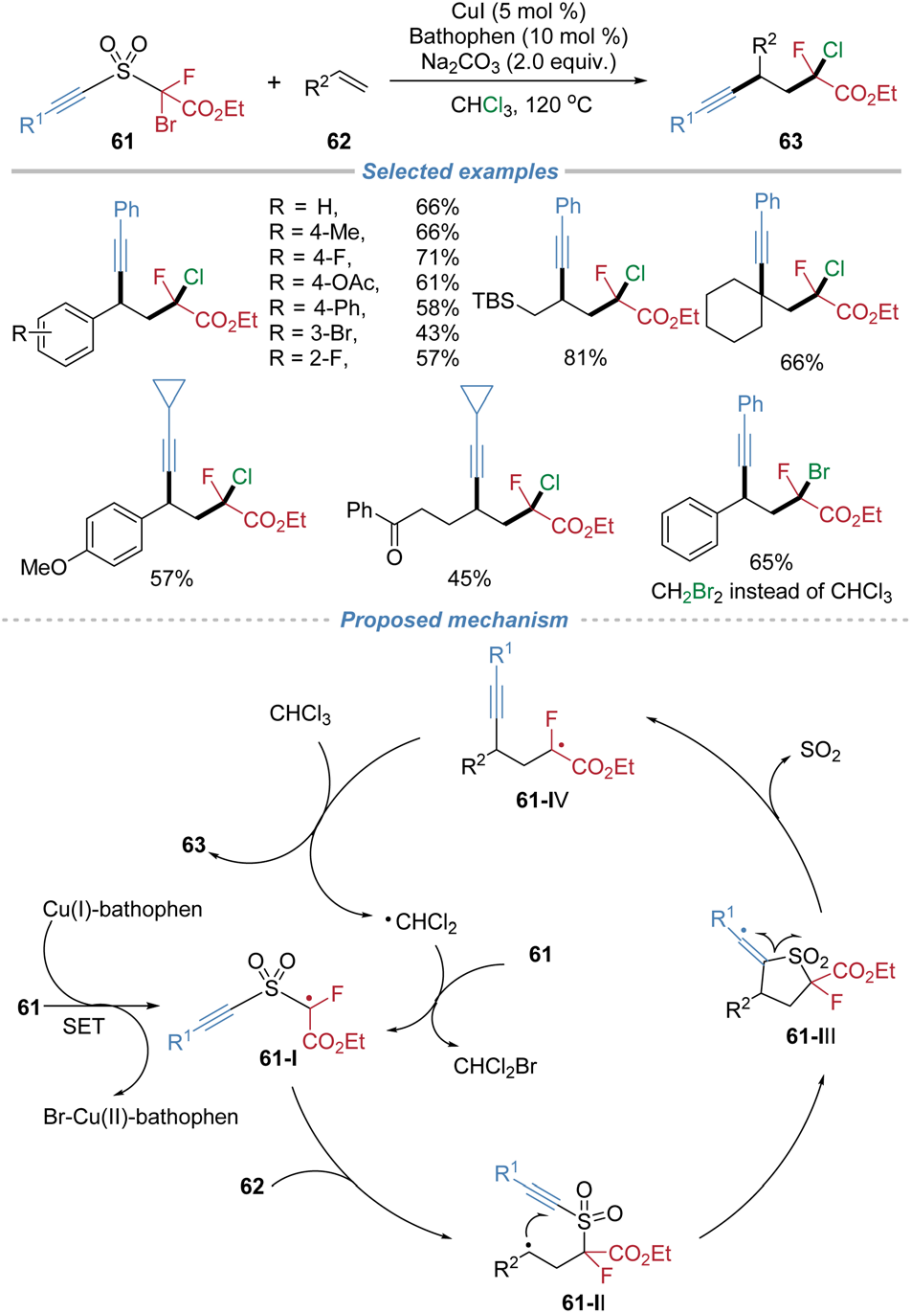
Radical monofluoroalkylative alkynylation of alkenes.

In 2019, our group disclosed a radical alkynylative functionalization of alkenes, achieving the concurrent incorporation of alkynyl and monofluoroalkyl groups (Scheme 22).^25^ This method employs the sulfone-based bifunctional reagents 61 featuring a bromofluoroacetate moiety as the radical docking site and an alkynyl as the migrating group. Under copper catalysis, the fluoroalkyl radical 61-I adds to the alkene, followed by an intramolecular migration of the alkynyl group *via* C–S bond cleavage and SO_2_ extrusion. The resulting radical intermediate 61-IV is subsequently terminated by a halogen atom abstracted from the solvent, completing a radical chain process. The reaction accommodates a broad range of alkene substrates, including both activated and unactivated alkenes. The *d*.*r*. is poor mainly because of the high reaction temperature and the terminating chlorine abstraction step proceeds without effective stereocontrol. This method provides efficient access to valuable monofluoroalkyl-substituted aliphatic alkynes, and exhibits considerable utility in the late-stage modification of complex molecules.

### 2.4 Oximino migration

**Scheme 23:**
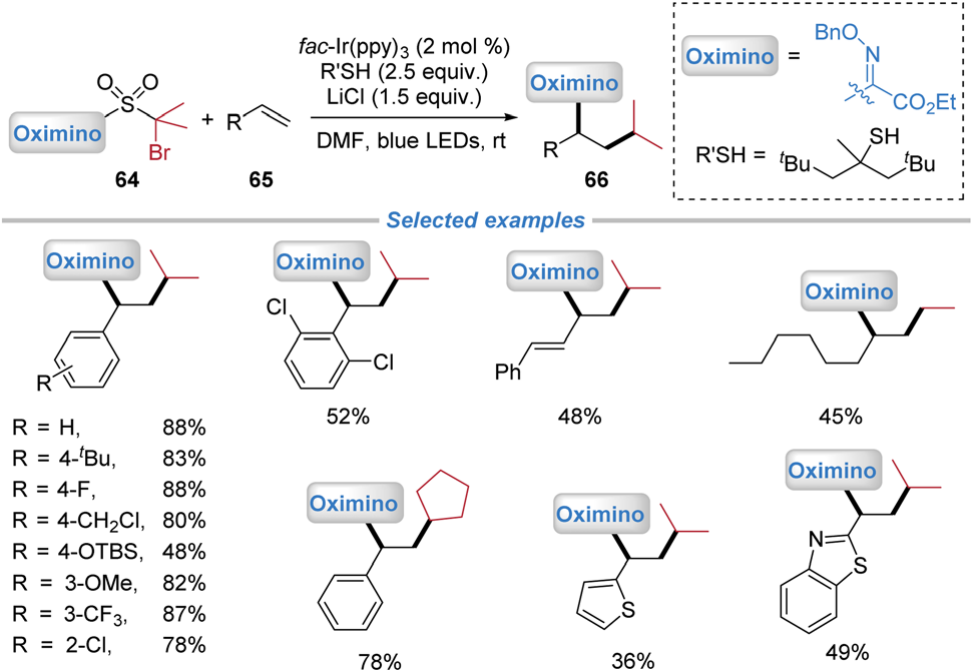
Polarity-umpolung oximino-alkylation of alkenes.

In 2020, our group demonstrated the versatility of the polarity umpolung strategy enabled by the docking–migration process, and extended the migration group from the heteroaryl to the oximino moiety (Scheme 23).^8^ Using the oximino-containing bifunctional sulfone reagents 64, the radical oximino-alkylation of both activated and unactivated alkenes was achieved under photoredox conditions. The addition of LiCl as a Lewis acid additive was found to significantly enhance the reaction efficiency, likely through activation of the oximino group. This transformation proceeded *via* a mechanism analogous to that of the heteroaryl migration shown in Scheme 4, involving radical addition to the alkene, intramolecular oximino migration, and SO_2_ extrusion, followed by reduction to yield the corresponding products.

### 2.5 Alkenyl migration

**Scheme 24:**
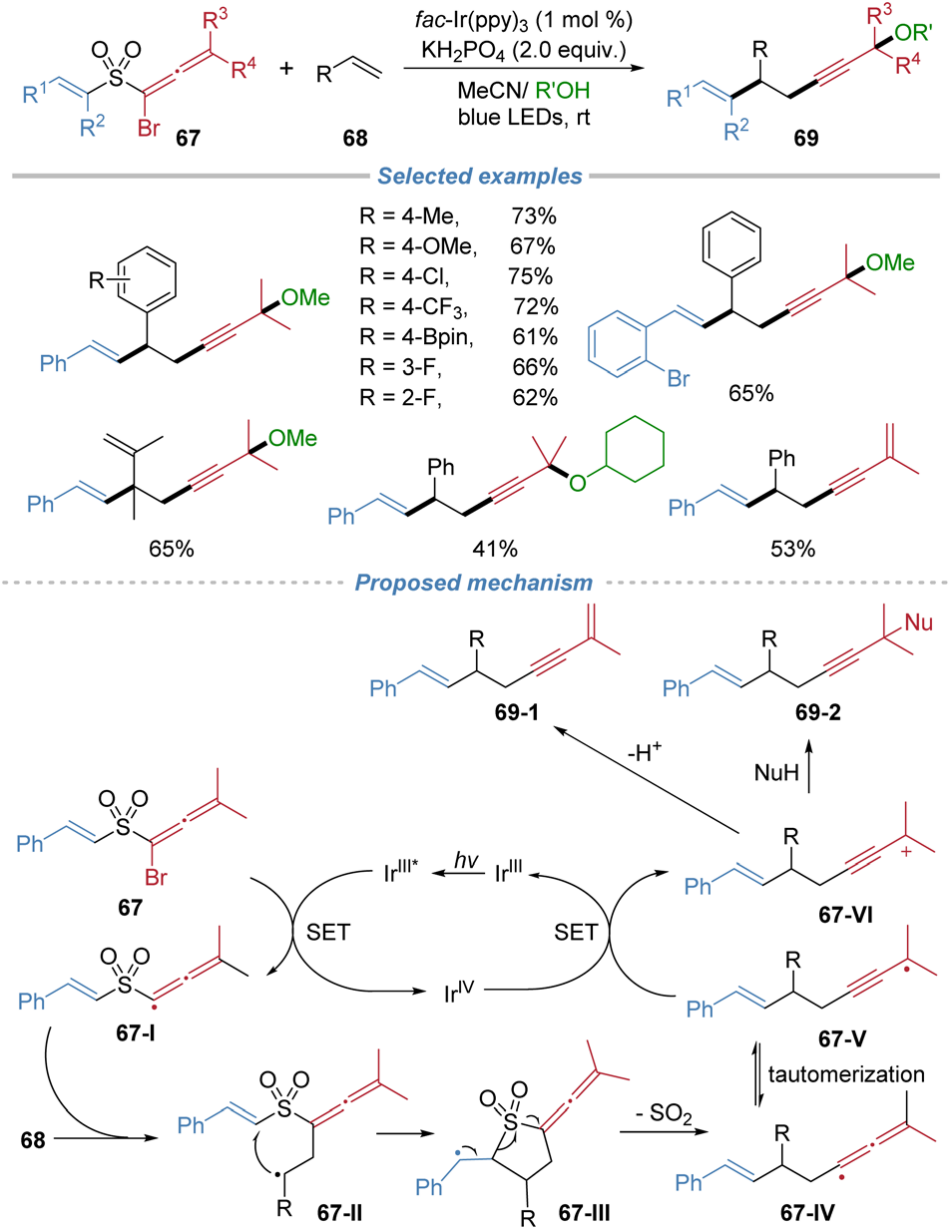
Radical 1,2-alkynylalkenylation and 1,2-enynylalkenylation of alkenes.

In 2021, our group applied the radical docking–migration strategy to concomitant installation of two unsaturated C–C bonds across alkenes (Scheme 24).^26^ The devised bifunctional sulfone reagents 67 bear a bromoallenyl group and an alkenyl migrating group. Under mild photocatalytic conditions, single-electron reduction of the reagent generates a sulfone-stabilized allenyl radical 67-I, which is sufficiently persistent to undergo intermolecular addition to alkenes to form the alkyl radical 67-II. This radical docking event is followed by an intramolecular alkenyl migration and SO_2_ extrusion cascade, delivering valuable 1,5-enyne products with exclusive stereoselectivity. The protocol exhibits broad substrate scope, encompassing styrenes, aliphatic alkenes, internal alkenes, and complex alkene molecules. By simply modulating the reaction solvent, the reaction was further extended to achieve 1,2-enynylalkenylation, providing direct access to densely functionalized enynes.

**Scheme 25:**
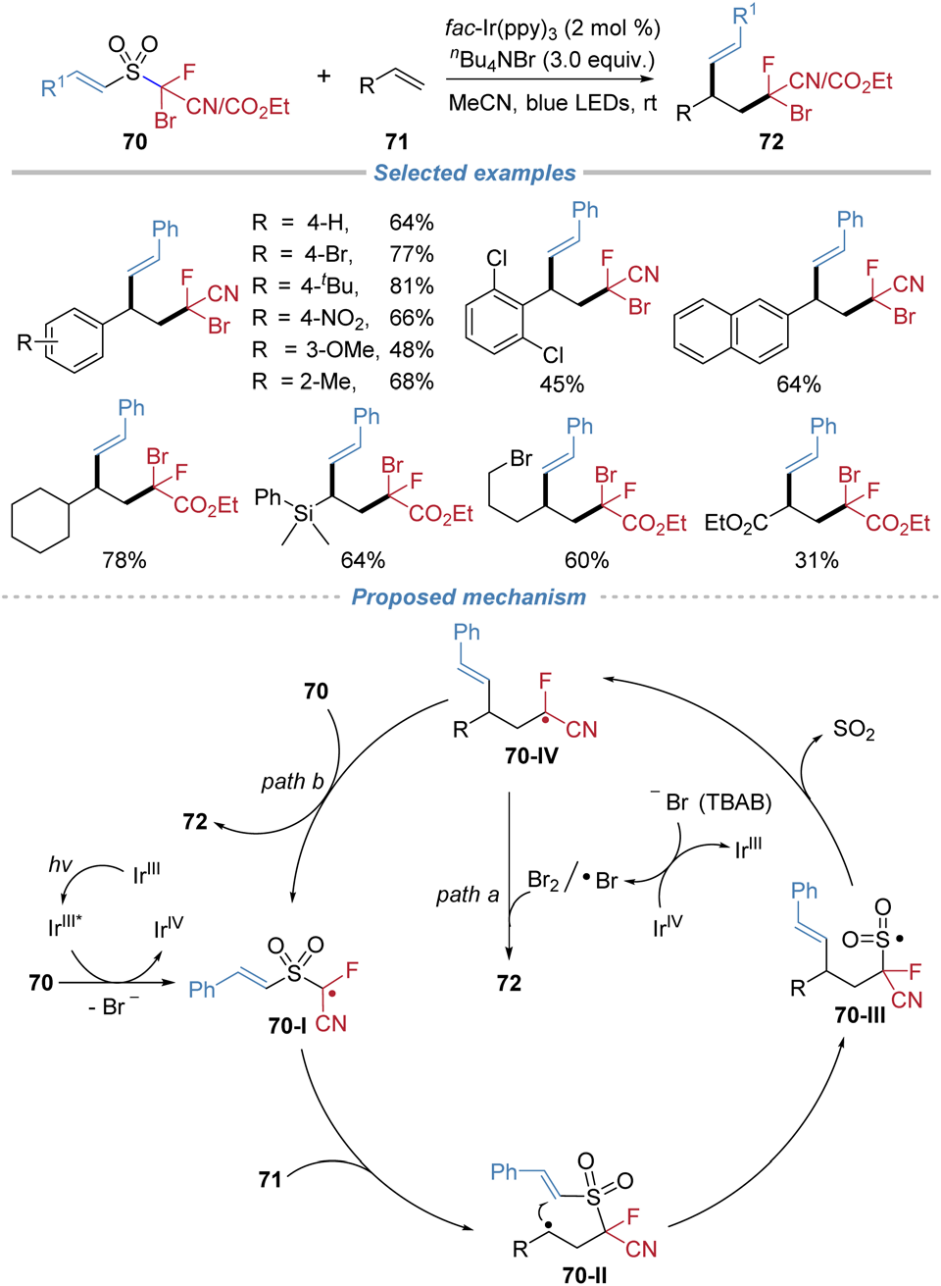
Radical fluoroalkylative alkenylation of alkenes.

The radical fluoroalkylative alkenylation of alkenes was also accomplished by using the docking–migration strategy (Scheme 25).^27^ This approach employed the bifunctional sulfone reagents 70 that contain both a bromofluoroalkyl group and an alkenyl migrating group, affording vinylated products exclusively as the *E*-isomer. Under mild photoredox catalysis with *fac*-Ir(ppy)_3_, the reaction is initiated *via* single-electron reduction of the C–Br bond, generating a fluoroalkyl radical 70-I. This radical adds to alkene, and the resulting carbon-centered radical 70-II undergoes an intramolecular cyclization with the tethered alkenyl sulfone moiety. This event triggers a sequence of alkenyl migration and SO_2_ extrusion, leading to the formation of the final product. The *d*.*r*. is generally low because the terminating bromine abstraction step proceeds without effective stereocontrol. The transformation accommodates a wide array of styrenes and aliphatic alkenes, as well as complex natural products and drug derivatives.

While the use of sulfone-based reagents enables remarkable transformations of both activated and unactivated alkenes, the extrusion of SO_2_ limits the atom economy of the reaction. Moreover, current asymmetric strategies predominantly rely on chiral sulfur auxiliaries (sulfoximines, sulfinamides), which are excellent for stereocontrol but require additional synthetic steps to install. A general, catalytic enantioselective platform using chiral catalysts remains unexplored.

## 3 Alkyne difunctionalization

### 3.1 (Hetero)Aryl migration

**Scheme 26:**
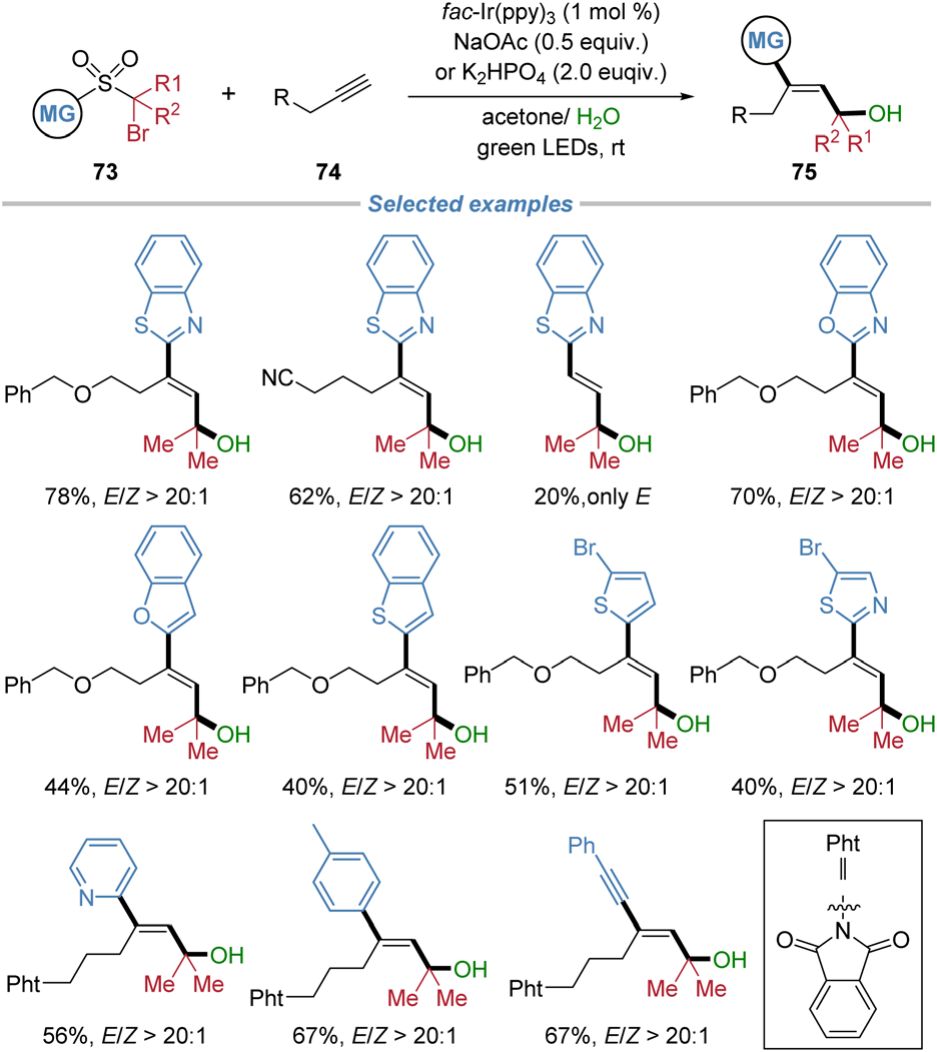
*E*-Selective radical difunctionalization of unactivated alkynes.

In 2024, our group reported the radical difunctionalization of unactivated aliphatic alkynes, achieving excellent chemo- and stereo-selectivity that have long remained elusive (Scheme 26).^28^ The photoredox-neutral cascade enables the concomitant incorporation of a hydroxyalkyl and a (hetero)aryl group across the alkyne, providing modular access to a diverse array of highly functionalized *E*-allylic alcohols 75. This method is noteworthy for its remarkable tolerance of aliphatic alkynes containing benzylic C–H bonds prone to intramolecular hydrogen atom transfer, a common side reaction that compromises selectivity in such transformations. The protocol exhibits broad functional group compatibility, encompassing a wide range of sensitive structural motifs, and its utility is highlighted by successful applications in the late-stage modification of complex natural products and pharmaceutical molecules.

**Scheme 27:**
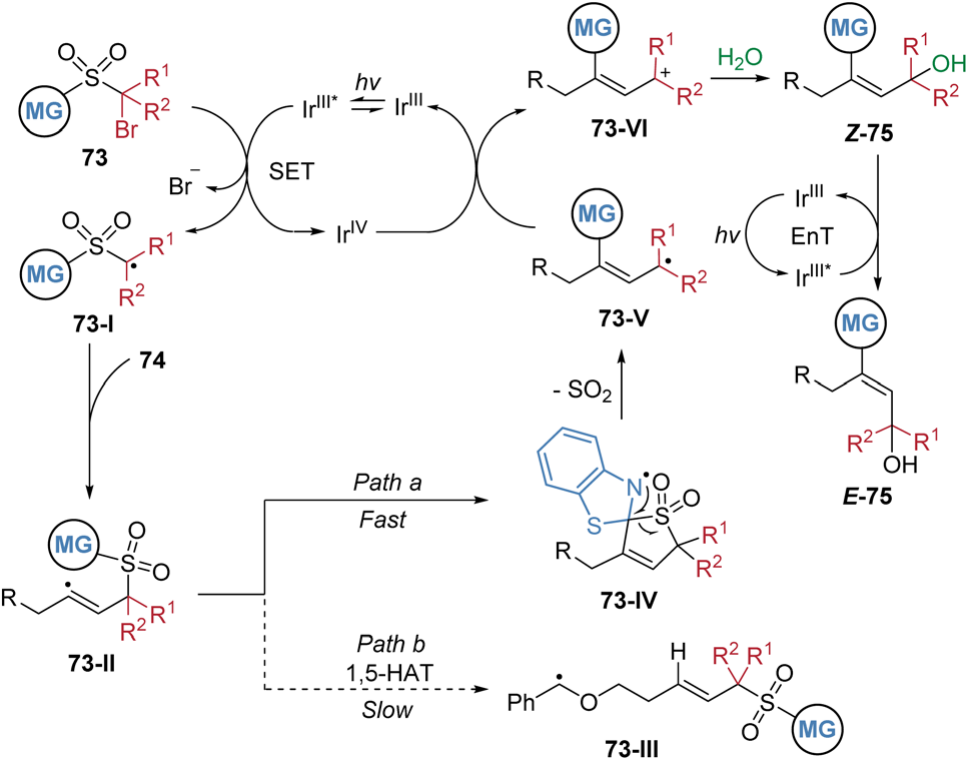
Proposed mechanism for radical difunctionalization of aliphatic alkynes.

The proposed mechanism is depicted in Scheme 27. Initially, single-electron transfer from photoexcited *fac*-Ir(ppy)_3_ to substrate 73 generates an alkyl radical intermediate 73-I, which undergoes addition to alkyne 74, yielding an alkenyl radical species 73-II. Cyclic voltammetry measurements of 73 confirmed that its C–Br bond is susceptible to reduction by the excited Ir^III^* complex. Subsequent intramolecular capture of the alkenyl radical by the heteroaryl group initiates the migration of the functional group. DFT calculations indicate that the competing 1,5-HAT pathway is energetically less favourable, consistent with the absence of such products under the reaction conditions. The 1,4-aryl migration occurs *via* a lower energy transition state compared to 1,5-HAT, leading to the more stable intermediate 73-IV. Extrusion of SO_2_ then affords radical species 73-V. This radical is then oxidized by the *in situ* generated Ir^IV^ species, which regenerates the ground-state Ir^III^ catalyst and forms cationic intermediate 73-VI. Nucleophilic addition of water to 73-VI gives rise to intermediate *Z*-75. Finally, in the presence of the photosensitizer, energy transfer facilitates isomerization of *Z*-75 to the thermodynamically stable product *E*-75.

**Scheme 28:**
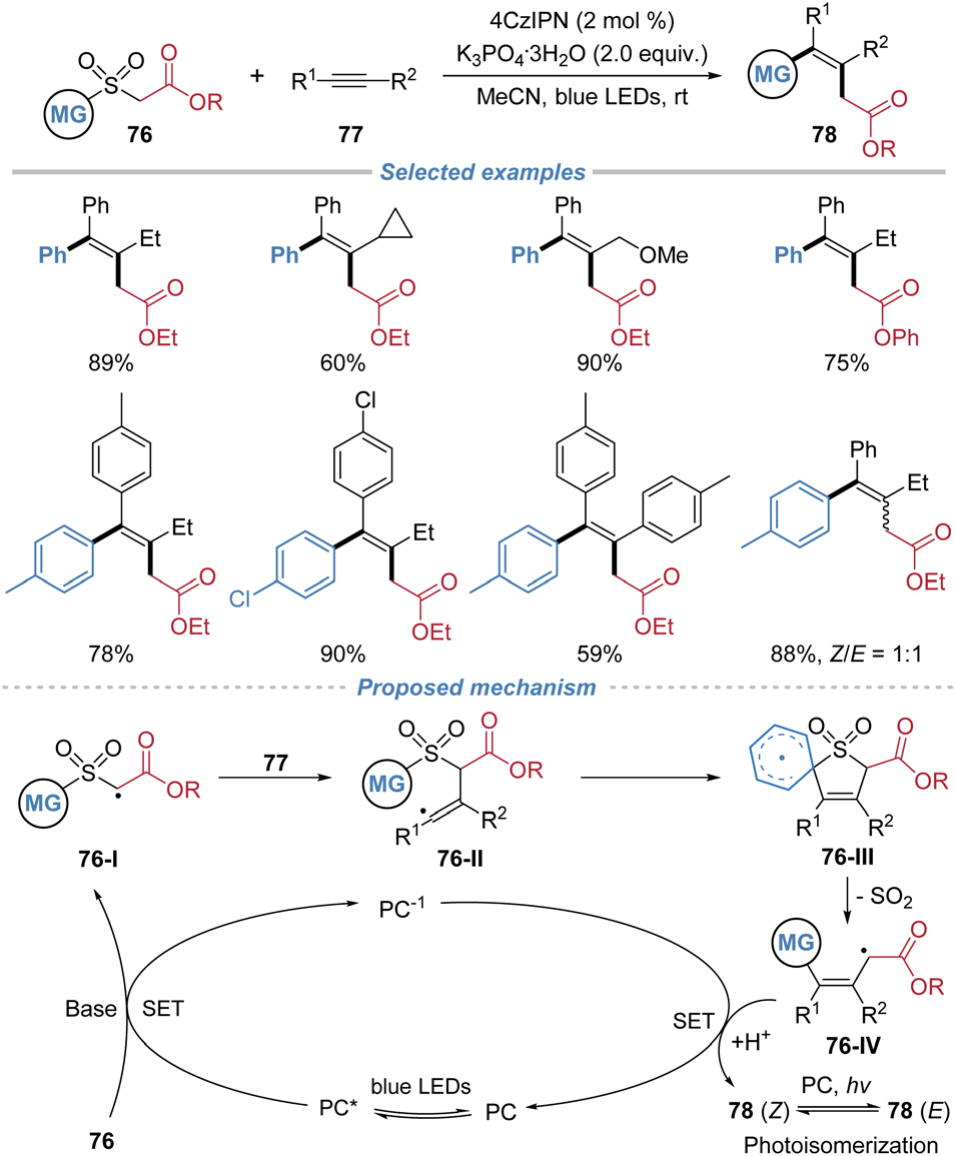
Radical alkylarylation of alkynes using arylsulfonyl acetates as bifunctional reagents.

In 2024, Liu *et al.* achieved the radical difunctionalization of internal alkynes by employing arylsulfonyl acetates 76 as bifunctional reagents (Scheme 28).^29^ Under photoredox catalysis, single-electron oxidation of the sulfonyl acetate reagent generates an electrophilic carbon-centered radical 76-I, which regioselectively adds to the alkyne, forming a vinyl radical intermediate 76-II. This species then undergoes intramolecular *ipso*-addition to the tethered (hetero)arene, triggering a sequential Smiles rearrangement with concomitant extrusion of SO_2_, which facilitates aryl migration and forges a new C–C bond. This cascade successfully installs both an alkyl carboxylate and an aryl group across the alkyne in a single operation, affording valuable all-carbon tetrasubstituted alkenes. The method exhibits broad substrate scope, excellent functional group tolerance, and applicability to both internal and terminal alkynes.

**Scheme 29:**
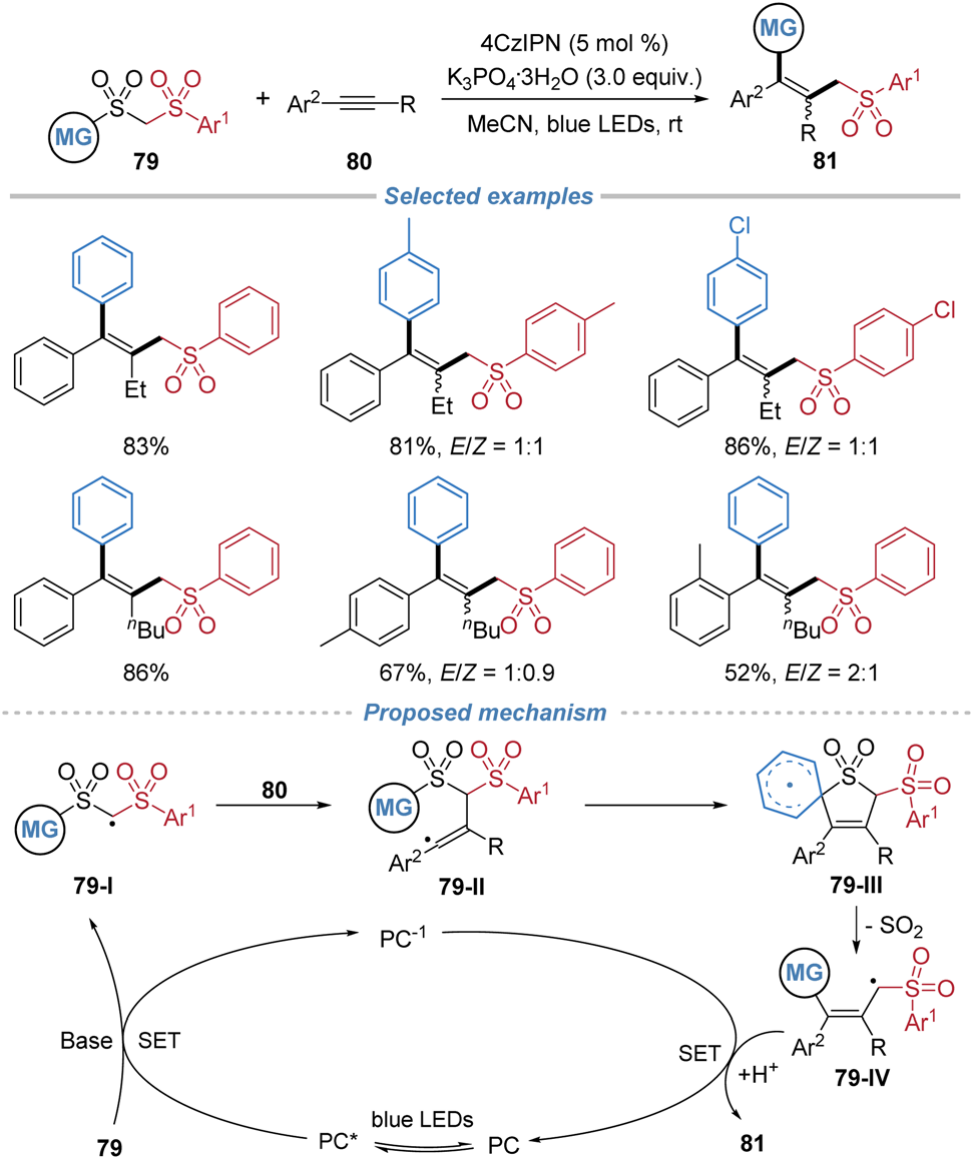
Radical alkylarylation of alkynes using bis-sulfone bifunctional reagents.

Subsequently, Liu *et al.* employed bis-sulfones 79 as bifunctional reagents in alkyne difunctionalization (Scheme 29).^30^ The reaction accommodates a range of substituted bis-sulfones and unsaturated substrates, including internal alkenes and alkynes, affording structurally diverse tetrasubstituted alkenes. However, the reaction proceeds without stereocontrol, leading to the formation of multi-substituted alkenes as *Z*/*E* mixtures. Mechanistic investigations, supported by cyclic voltammetry and Stern–Volmer quenching studies, indicate that the process begins with deprotonation of the bis-sulfone 79 to form an anionic intermediate. This species undergoes single-electron oxidation by the photoexcited 4CzIPN catalyst to generate an alkyl radical 79-I. Subsequent addition of 79-I across the alkyne produces radical intermediate 79-II, which engages in an intramolecular *ipso*-cyclization to form spirocyclic species 79-III. Fragmentation of 79-III with concomitant SO_2_ extrusion yields a new alkyl radical 79-IV, which is then reduced and protonated to furnish the final product while regenerating the ground-state photocatalyst.

**Scheme 30:**
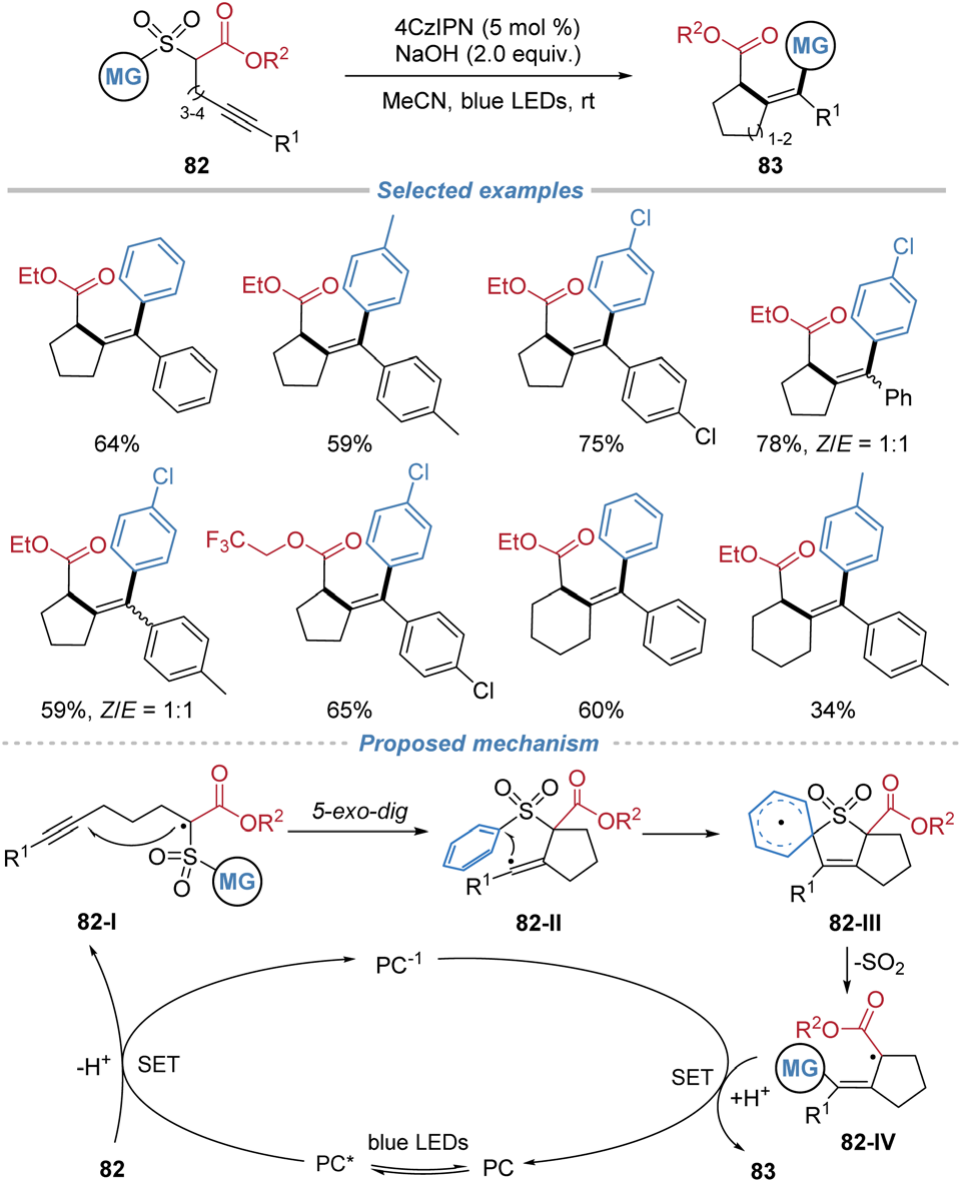
Photocatalytic Conia-ene reaction/Smiles rearrangement cascade.

Later, Liu *et al.* demonstrated an extension to intramolecular alkyne cyclization, employing alkyne-tethered α-sulfonyl esters 82 as substrates (Scheme 30).^31^ Under photoredox catalysis, single-electron oxidation generates a carbon-centered radical 82-I, which undergoes regioselective 5-*exo-dig* cyclization onto the alkyne, forming a vinyl radical intermediate 82-II. This species triggers an intramolecular Smiles rearrangement, facilitating aryl migration from the sulfonyl to the vinyl carbon with simultaneous extrusion of SO_2_, thereby forging a new C–C bond. This cascade effectively bypasses the traditional 1,5-hydrogen shift of classical Conia-ene reaction, enabling the direct synthesis of cyclic tetrasubstituted alkenes. The method exhibits broad substrate scope, good functional group tolerance, and is applicable to the construction of both five- and six-membered carbocycles as well as lactone-fused systems. The utility of this method is highlighted by its ability to efficiently cyclize low-reactivity substrates that are recalcitrant to conventional Conia-ene conditions.

**Scheme 31:**
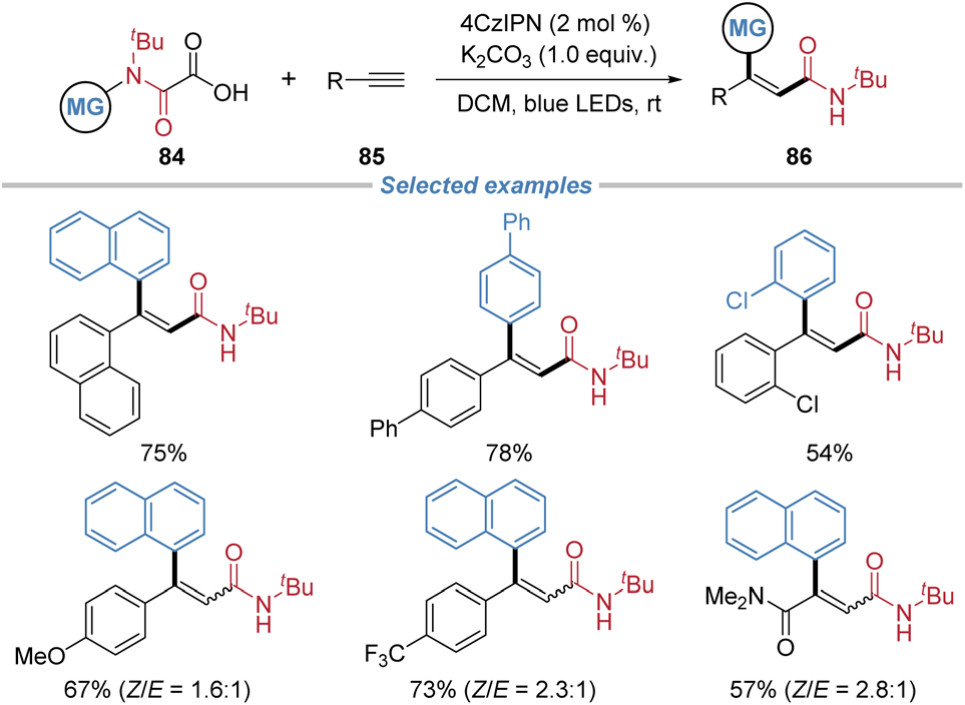
Radical carbamoylarylation of alkynes with *N*-aryl oxamic acids.

Kuninobu *et al.* demonstrated the utility of *N*-aryl oxamic acids 84 as bifunctional reagents for the radical carbamoylarylation of terminal alkynes under visible-light irradiation (Scheme 31).^32^ This approach generates a carbamoyl radical *via* decarboxylation, which then adds to alkynes. The resulting vinyl radical intermediate undergoes a radical-mediated 1,4-aryl migration *via* cleavage of the C–N bond, simultaneously forging a new C–C bond and delivering valuable β,β-diarylacrylamide products in moderate to good yields. The reaction exhibits a broad substrate scope, accommodating various *N*-aryl oxamic acids and arylacetylenes bearing both electron-donating and withdrawing groups.

**Scheme 32:**
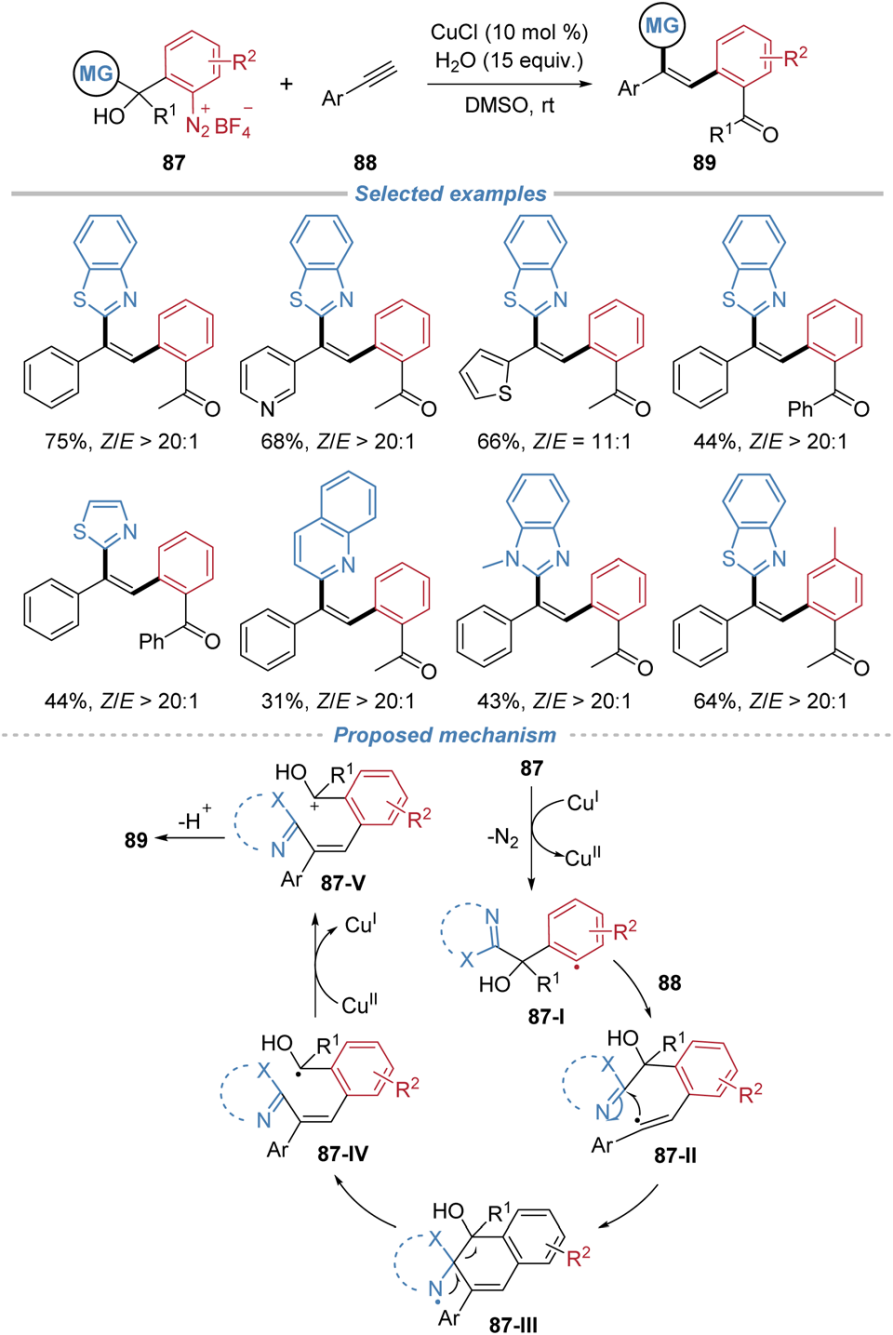
*Z*-Selective radical arylheteroarylation of alkynes.

Our group disclosed a *Z*-selective radical arylheteroarylation of aromatic alkynes using tertiary alcohol-substituted aryldiazonium salts 87 as bifunctional reagents (Scheme 32).^33^ This process employs cuprous chloride to reduce the diazonium salt, generating an aryl radical 87-I that adds to the alkyne. The resulting vinyl radical 87-II undergoes intramolecular cyclization followed by a 1,5-heteroaryl migration *via* β-scission of a C–C bond, leading to the formation of triarylethylenes with exceptional *Z*-selectivity. The method is applicable to a wide range of aromatic and heteroaromatic alkynes, including complex drug derivatives. The reaction proceeds efficiently on a gram scale and the products serve as versatile intermediates for further transformations, including the synthesis of a novel spin-trapping reagent capable of detecting methyl radicals.

### 3.2 Imino migration

**Scheme 33:**
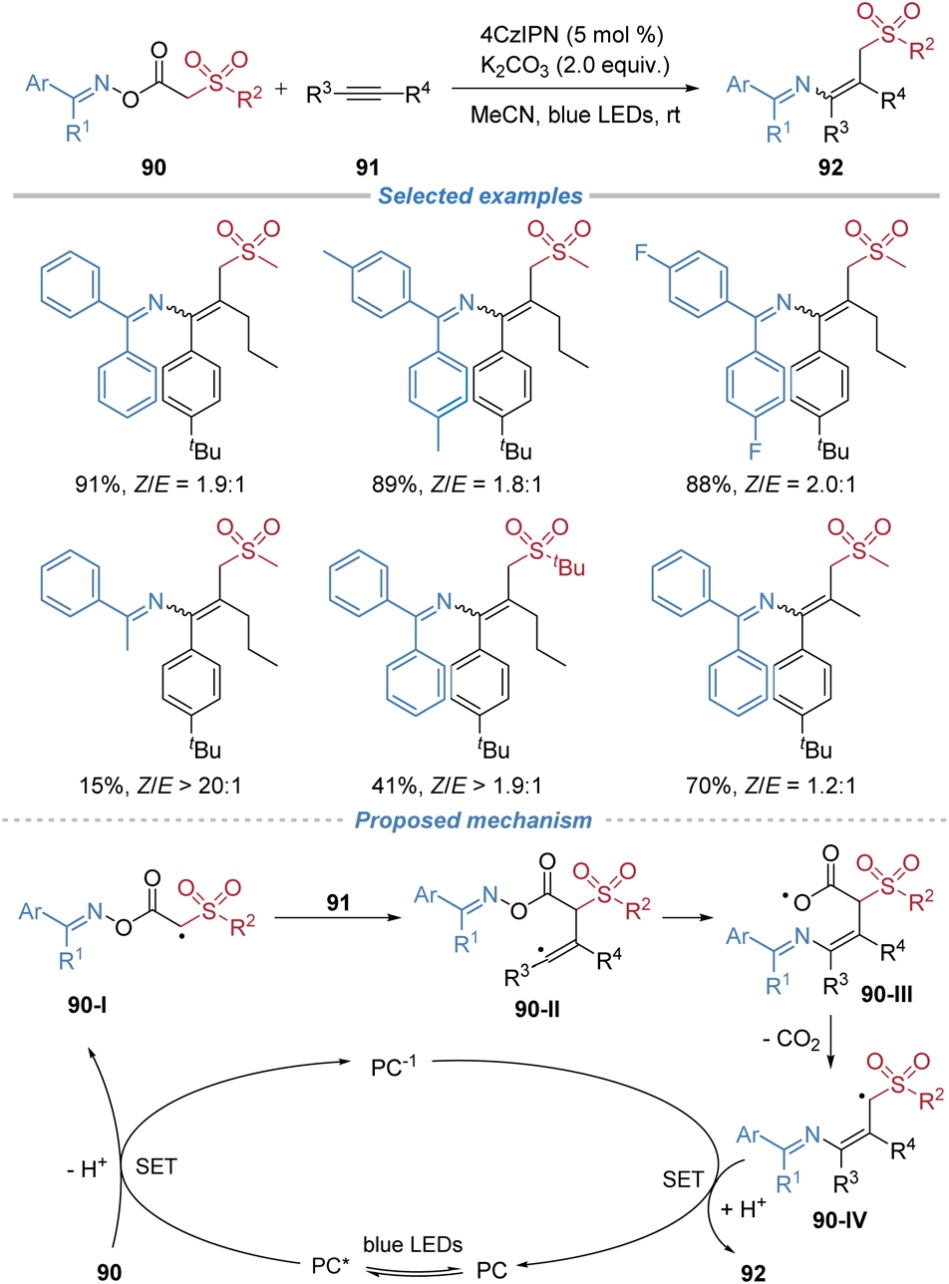
Oxime esters as bifunctional reagents for radical alkylamination of alkynes.

Liu *et al.* reported the use of oxime esters 90 as bifunctional reagents for the radical alkylamination of alkynes under photoredox conditions (Scheme 33).^34^ This reaction proceeds *via* a sequence involving single-electron oxidation of the reagent, generating a carbon-centered radical 90-I that adds across the alkyne. The resulting vinyl radical 90-II undergoes 6-*exo-trig* cyclization, followed by β-scission of the N–O bond and decarboxylation, leading to migration of the imino group and formation of tetrasubstituted alkenes. However, the stereoselectivity of the alkene products is not satisfactory. The method is amenable to gram-scale synthesis. Additionally, the products serve as versatile intermediates for further transformations, including hydrolysis to ketones and cyclization to polysubstituted pyrroles.

Achieving high stereoselectivity in alkyne difunctionalization, as exemplified here, is a landmark achievement. However, a pervasive challenge across many radical alkyne reactions is the control of *Z*/*E* selectivity. While docking–migration can enforce specific geometries, many systems still yield mixtures.

## 4 Miscellaneous

### 4.1 Difunctionalization of enamine intermediates

**Scheme 34:**
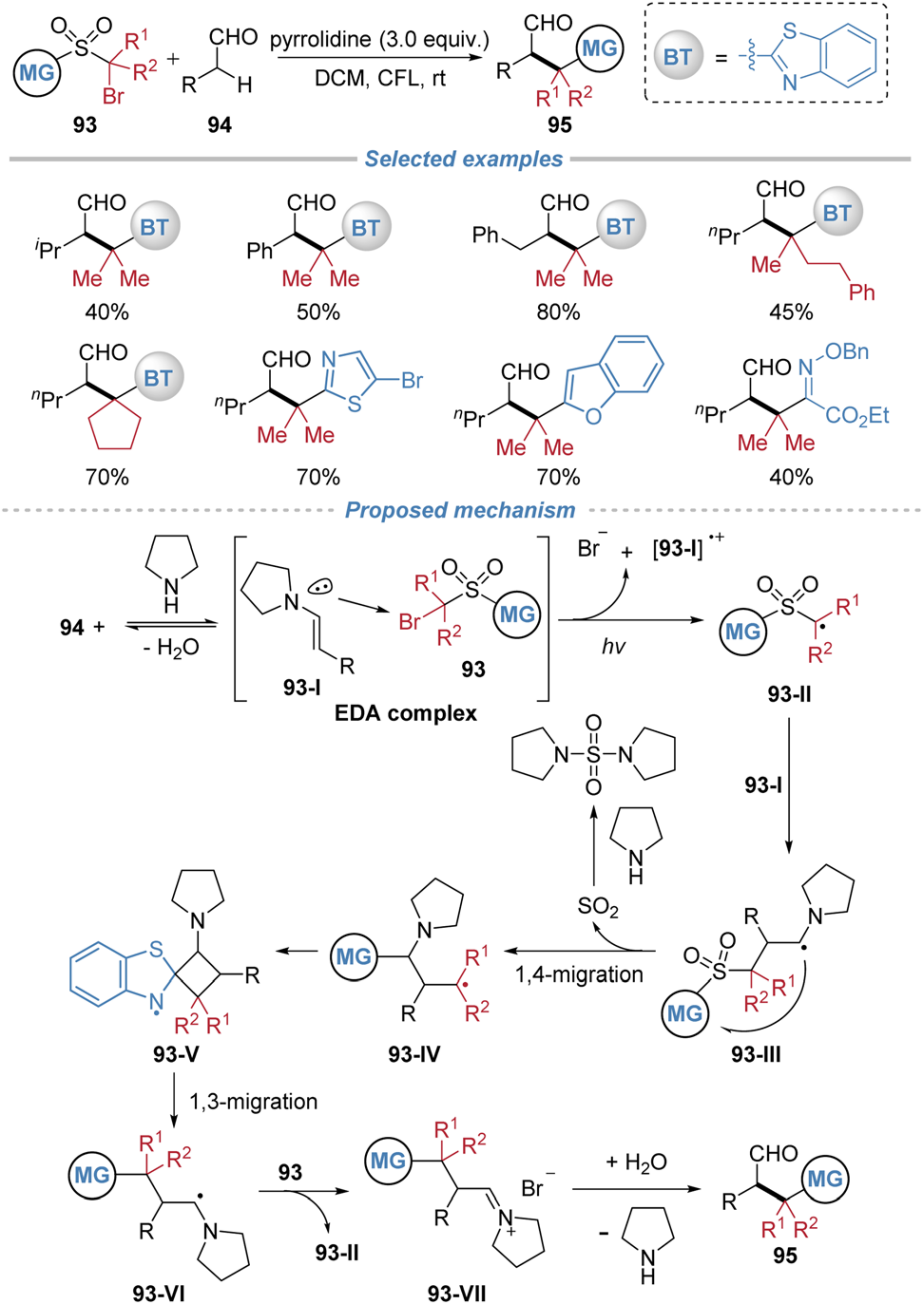
Radical-mediated α-*tert*-alkylation of aldehydes *via* consecutive heteroaryl migrations.

Our group disclosed a radical-mediated α-*tert*-alkylation of aliphatic aldehydes *via* consecutive 1,4- and 1,3-heteroaryl migrations, providing an efficient route to structurally complex aliphatic aldehydes under metal-free photochemical conditions (Scheme 34).^35^ The reaction is initiated by the formation of an electron donor–acceptor (EDA) complex between an *in situ*-generated enamine intermediate and an α-bromo sulfone, which upon photoexcitation generates a radical species. This intermediate undergoes a sequence of 1,4- and 1,3-heteroaryl migrations, ultimately leading to the installation of a tertiary alkyl group at the α-position of the aldehyde. Mechanistic studies, including radical trapping experiments, crossover tests, and DFT calculations, support a radical chain process involving intramolecular migration steps. Mechanistic investigations delineate the following sequence: initially, condensation of the aldehyde 94 with a secondary amine generates an enamine 93-I, which associates with the α-bromo sulfone reagent 93 to form an electron donor–acceptor complex. Photoexcitation of this assembly triggers a single-electron transfer, furnishing an α-sulfonyl alkyl radical 93-II and an enamine-derived radical cation. The electrophilic carbon-centered radical is then captured by the nucleophilic enamine, forming a new C–C bond and generating the radical species 93-III. This radical adduct subsequently extrudes SO_2_*via* a 1,4-group migration. The resulting alkyl radical 93-IV then undergoes a less common 1,3-heteroaryl shift, proposed to proceed *via* a constrained four-membered ring transition state, to deliver a stabilized α-amino radical 93-VI. Finally, this intermediate is oxidized by the α-bromo sulfone 93, regenerating the initial radical species 93-II to propagate the chain and yielding an iminium ion 93-VII, which upon hydrolysis furnishes the observed α-tertiary aldehyde product.

### 4.2 Difunctionalization of heteroarenes and polycyclic aromatic hydrocarbons

**Scheme 35:**
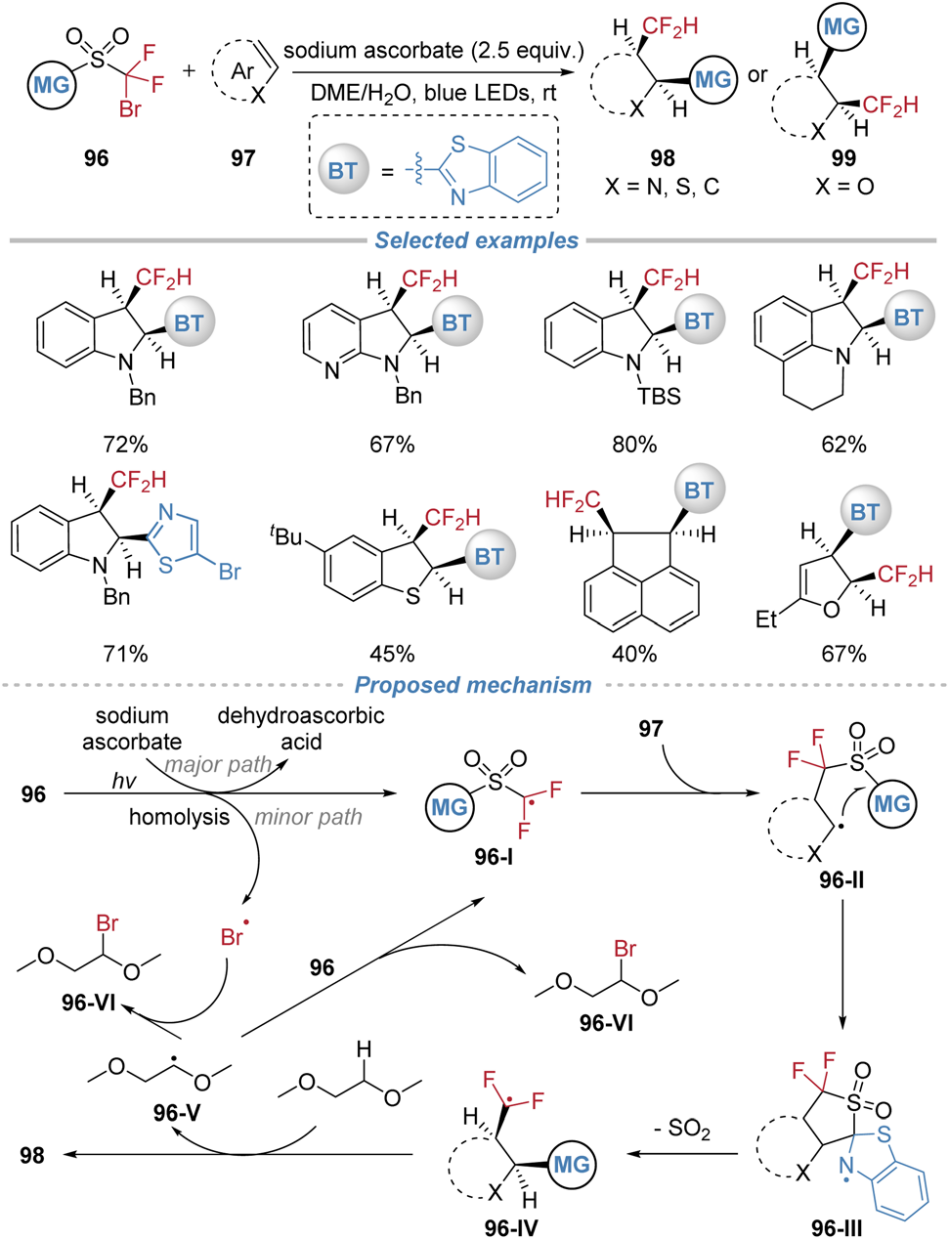
*cis*-Difluoromethyl hetarylative dearomatization.

Recently, our group also achieved an uncyclized *cis*-selective difluoromethyl hetarylation of heteroarenes under mild photochemical conditions without an external photocatalyst (Scheme 35).^36^ This method utilizes bifunctional sulfone reagents 96 to concomitantly install CF_2_H and hetaryl groups across the C=C bonds of indoles, benzothiophenes, furans, thiophenes, and a few polycyclic aromatic hydrocarbons. The transformation proceeds under blue light irradiation in a DME/H_2_O solvent using sodium ascorbate as the reductant. The mechanism is proposed to initiate *via* single-electron transfer between the sulfone reagent and sodium ascorbate, generating an electrophilic difluoroalkyl radical 96-I. This radical adds to the heteroarene, forming an adduct that undergoes an intramolecular heteroaryl migration through a kinetically favored five-membered cyclic transition state. Subsequent extrusion of SO_2_ delivers a difluoromethyl radical 96-IV, which abstracts a hydrogen atom from the solvent to furnish the final *cis*-product. The newly formed radical 96-V abstracts Br atom from the sulfone reagent, regenerating intermediate 96-I to propagate the radical chain. This method features broad substrate scope, excellent functional group tolerance, and high stereoselectivity, providing direct access to thermodynamically disfavored *cis*-difunctionalized (hetero)aryl derivatives.

## 5 Summary and outlook

The radical docking–migration strategy has established itself as a powerful and versatile platform for the difunctionalization of alkenes and alkynes, enabling the concomitant incorporation of two distinct functional groups across unsaturated carbon–carbon bonds with high levels of regio- and stereoselectivity. By employing rationally designed bifunctional reagents derived from sulfones, tertiary alcohols, diaryl ethers, sulfinamides, and sulfoximines, this approach proves applicable to both activated and unactivated alkenes and alkynes. The modular nature of these reagents permits the introduction of diverse migrating groups—such as aryl, heteroaryl, alkynyl, oximino, alkenyl, and imino moieties—thereby facilitating efficient access to a broad spectrum of complex molecular architecture from simple starting materials.

Notably, asymmetric versions of these transformations have been realized through the use of chiral sulfoximines and sulfinamides as bifunctional reagents, enabling the construction of enantioenriched scaffolds. Mechanistic investigations have shed light on key steps, including radical addition, migration, and termination, often corroborated by DFT calculations and spectroscopic evidence, thereby affirming the robustness of this strategy.

### 5.1 Design principles of bifunctional reagents

The use of sulfonyl as the linker in radical-mediated Smiles rearrangements offers unique advantages. The first advantage lies in the exergonic driving force provided by the extrusion of gaseous SO_2_, which propels the migration process. Concurrently, the sulfonyl group enables kinetically favored 5-membered cyclic transition states for the intramolecular migration and inverts the polarity of the initial radical, making it electrophilic and thus compatible with unactivated alkenes. Furthermore, sulfonyl acts as a traceless linker, exiting cleanly as a gas and leaving no residue in the product. In addition, when employed as linkers, both chiral aryl sulfinamides and chiral sulfoximines utilize their stereogenic sulfur (IV or VI) center as a temporary chiral auxiliary. During the radical Smiles rearrangement, chiral information is transferred from this sulfur atom to a new carbon stereocenter in the product, typically with high stereospecificity.

### 5.2 Synthetic accessibility of bifunctional reagents

Although most of the bifunctional reagents described herein are not commercially available, their synthetic routes have been systematically optimized, allowing most to be efficiently prepared in 2–4 steps from readily available starting materials. Although the synthetic efforts to access bifunctional reagents is a disadvantageous factor, this is offset by the high-value, complex products obtained in a single operation.

### 5.3 Reasons for the choice of the catalytic system

The choice of catalytic system is dictated by the electronic properties of the bifunctional reagents and the desired reaction pathways. Photoredox catalysis is predominantly employed to generate radical species from C–Br and C–I bonds under mild conditions *via* SET. Copper catalysis is often selected to mediate halogen atom transfer (XAT) or SET processes, particularly when involving diazonium salts or facilitating radical chain propagations. EDA complex photoactivation provides a metal- and external photocatalyst-free pathway, ideal for generating radicals from reagents with matched redox potentials. Finally, catalyst-free conditions are viable when the reagent itself (*e.g.*, diazonium salts) can serve as a radical precursor upon photoexcitation.

### 5.4 Outlook

Despite rapid progress in this area, several challenges and opportunities remain. First, the development of non-sulfone-based linkers is highly desirable to broaden reagent diversity and enhance functional group compatibility. Second, although asymmetric variants have been achieved using chiral sulfur auxiliaries, general catalytic enantioselective protocols—particularly those employing chiral photocatalysts or Lewis acids—remain underdeveloped. Third, asymmetric migration of other valuable groups, such as cyano, ester, and phosphorus-based functionalities, remains largely unexplored and constitutes a promising direction for future research. Finally, the application of this methodology to the synthesis of complex functional molecules—including natural products, pharmaceuticals, and polymeric materials—represents an important and anticipated future endeavour.

## Author contributions

Z. C. and F. C. prepared the draft. C. Z. developed the concept and revised this review.

## Conflicts of interest

There are no conflicts to declare.

## Data Availability

No primary research results, software or code have been included and no new data were generated or analysed as part of this review.
